# Structural roles of PCV2 capsid protein N-terminus in PCV2 particle assembly and identification of PCV2 type-specific neutralizing epitope

**DOI:** 10.1371/journal.ppat.1007562

**Published:** 2019-03-01

**Authors:** Xiaobing Mo, Xiangdong Li, Bo Yin, Junhua Deng, Kegong Tian, Adam Yuan

**Affiliations:** 1 Department of Biological Sciences and Centre for Bioimaging Sciences, National University of Singapore, Singapore; 2 National Research Center for Veterinary Medicine, Luoyang, Henan, China; 3 National University of Singapore (Suzhou) Research Institute, Suzhou, Jiangsu, China; 4 College of Animal Science and Veterinary Medicine, Henan Agricultural University, Zhengzhou, Henan, China; Penn State University School of Medicine, UNITED STATES

## Abstract

Postweaning multisystemic wasting disease (PMWS) in piglets caused by porcine circovirus type 2 (PCV2) is one of the major threats to most pig farms worldwide. Among all the PCV types, PCV2 is the dominant genotype causing PMWS and associated diseases. Considerable efforts were made to study the virus-like-particle (VLP) assembly and the specific PCV2-associated epitope(s) in order to establish the solid foundation for engineered PCV2 vaccine development. Although the N-terminal fragment including Nuclear Localization Signal (NLS) sequence seems important for recombinant PCV2 capsid protein expression and VLP assembly, the detailed structural and functional information regarding this important fragment are largely unknown. In this study, we report crystal structure of PCV2 VLP assembled from N-terminal NLS truncated PCV2 capsid protein at 2.8 Å resolution and cryo-EM structure of PCV2 VLP assembled from full-length PCV2 capsid protein at 4.1Å resolution. Our *in vitro* PCV2 VLP assembly results show that NLS-truncated PCV2 capsid protein only forms instable VLPs which were easily disassembled in solution, whereas full-length PCV2 capsid protein forms stable VLPs due to interaction between ^15^PRSHLGQILRRRP^27^ **(α-helix)** and ^33^RHRYRWRRKN^42^ **(NLS-B)** in a repeated manner. In addition, our results also showed that N-terminal truncation of PCV2 capsid protein up to 27 residues still forms PCV2 particles in solution with similar size and immunogenicity, while N-terminal truncation of PCV2 capsid protein with more than 30 residues is not able to form stable PCV2 particles in solution, demonstrating the importance of interaction between the α-helix at N-terminal and NLS-B in PCV2 VLP formation. Moreover, we also report the cryo-EM structure of PCV2 VLP in complex with 3H11-Fab, a PCV2 type-specific neutralizing antibody, at 15 Å resolution. MAb-3H11 specifically recognizes one exposed epitope located on the VLP surface EF-loop (residues 128–143), which is further confirmed by PCV1-PCV2 epitope swapping assay. Hence, our results have revealed the structural roles of N-terminal fragment of PCV2 capsid protein in PCV2 particle assembly and pinpointed one PCV2 type-specific neutralizing epitope for the first time, which could provide clear clue for next generation PCV2 vaccine and diagnostic kits development.

## Author summary

Porcine circovirus type 2 (PCV2) is considered as one of the most wide-spread pathogens threatening swine production by causing postweaning multisystemic wasting disease (PMWS) in piglets worldwide. Several VLP-based PCV2 vaccines are commercially available which significantly reduce the viral burden and virally induced lesions. However, prophylactic efficacy of VLP-based PCV2 vaccine largely relies on the correct VLP assembly from the individual PCV2 capsid protein. Notably, limited structural information of PCV2 N-terminal fragment containing arginine-rich patches significantly delays our understanding of PCV2 assembly at the molecular level, and the lack of solid evidence in identification of PCV2 type-specific epitope delays the development of PCV2 type-specific diagnosis kits. In this study, through the combination of structural and immunological approaches, we are able, for the first time, to disclose the structural details of the N-terminal Nuclear Localization Signal (NLS) region of PCV2 capsid protein. We show that the interaction between the α-helix from one capsid protein and the NLS-B from an adjacent capsid protein within the pentamer stabilizes the assembled PCV2 VLP in solution. Moreover, by the combination of structural determination and biochemical mapping, we have identified that a short linear sequence (^134^KATALT^139^) located within PCV2 EF-loop is a unique PCV2 type-specific neutralizing epitope. Therefore, our work has revealed the detailed structural information of PCV2 particle assembly and a PCV2 type-specific neutralizing epitope, which should provide insightful information for virus-host interaction studies and next-generation PCV2 vaccine and type-specific diagnostic kits development.

## Introduction

In early 1990s, a postweaning multisystemic wasting syndrome (PMWS) outbreak in Western Canada [1] was reported due to the spreading of a small, non-enveloped virus containing a single-stranded, circular DNA genome, which was later diagnosed as the porcine circovirus (PCV) [2]. Subsequently, PMWS and PCV pathogens were identified in pigs in USA, China and other countries [3]. PCV2 belongs to the family of Circoviridae containing several types, such as PCV1 and PCV2 [4]. Currently, PCV2 is the dominant pathogen causing PMWS and a number of associated diseases in pigs [5–7], severely affecting swine production worldwide [8–15]. Remarkably, PCV seems to continue evolving to yield more pathogenic genotypes probably due to selection pressure. Initially, only PCV1 and PCV2 (PCV2a, PCV2b, PCV2c, PCV2d, PCV2e and PCV2f) were identified, however a distantly related porcine circovirus (PCV3) were recently diagnosed both in US and China [16,17]. Among them, PCV1 is characterized as a non-pathogenic virus, PCV2 is identified as a causative agent for severe economic losses in the swine industry, whereas PCV3 is found to be associated with porcine dermatitis nephropathy syndrome (PDNS), reproductive failure, etc [18,19]. Among them, PCV2b genotype was the most widely spread PCV pathogen worldwide, whereas PCV2d has become a predominant PCV2 pathogen during the global PCV2 genotype shift recently [20–23]. Notably, PCV2 capsid protein (233/234 amino acids) encoded by the open reading frame 2 (ORF2) of PCV2 is capable of self-assembly into VLP and resembling the icosahedral morphology of the native PCV2 virions [24]. Serological results show that neutralization antibodies with high titers eliciting from the *in vitro* assembled PCV2 VLPs provide strong protection for piglets from homologous genotype PCV2 infection [25]. In the market, the engineered PCV2 vaccine, Ingelvac CircoFLEX, developed from the self-assembled VLP from PCV2 capsid protein shows superior prophylactic efficacy compared to the traditional vaccines created from inactivated PCV2 viruses [26–28].

In literature, the crystal structure of *in vitro* assembled PCV2 VLP derived from N-terminally truncated PCV2 capsid protein was determined at 2.3 Å resolution, whereas the cryo-EM structures of *in vitro* assembled full-length PCV2 VLP was determined at 9.6 Å resolution [29]. These structures show that PCV2 VLPs are assembled from 60 copies of PCV2 capsid proteins with the icosahedral symmetry and several exposed loops located on the viral surface, which may serve as the immunodominant epitopes eliciting neutralization antibodies [29,30]. Although these structures have provided a starting point to understand the structural principles of PCV2 VLP assembly and neutralization epitope identification, two critical questions remain largely unanswered: 1) the role of the N-terminal fragment of PCV2 capsid protein, including the nuclear localization signal (NLS) in PCV2 VLP assembly, and 2) the structural epitopes that differentiate PCV1 and PCV2 genotypes.

In this study, we sought to explore the structural roles of N-terminal fragments of PCV2 capsid protein in PCV2 assembly and investigate the structural basis of a PCV2 type-specific epitope. We determined the cryo-EM structure of non-tagged full length PCV2 capsid protein and the crystal structure of N-terminal His-tagged 1–45 residues truncated PCV2 capsid protein (PCV2-His-Δ45). Through the analysis and comparison of these two structures, we speculated that the interaction between the two N-terminal fragments of the PCV2 capsid protein (^15^PRSHLGQILRRRP^27^/α-helix and ^33^RHRYRWRRKN^42^/NLS-B) plays a significant role in stabilizing the assembled PCV2 VLPs in solution. The systematic truncation results validated our assumption. Additionally, we determined the cryo-EM structure of the full-length PCV2 VLP in complex with the Fab fragment of a PCV2 type-specific neutralizing monoclonal antibody, in which the structural docking model clearly shows that the CDR regions of mAb-3H11 Fab bind to a protruding EF-loop region (^134^KATALT^139^) located on the PCV2 VLP surface. This EF-loop region (^134^KATALT^139^) could serve as a PCV2 type-specific neutralizing epitope. Our results could provide insightful information for next generation PCV2 vaccine and diagnostic kit development.

## Results

### PCV2 VLP assembly from PCV2 capsid proteins

The full-length PCV2 capsid protein was expressed in *E*. *coli* and purified following a standard protocol developed in our lab [31]. The purity of the capsid protein can reach at least 95% **(S1A Fig)**. The purified protein samples were used for PCV2 VLP assembly checked by transmission electron microscopy and dynamic light scattering assays. Under transmission electron microscopy, homogeneous PCV2 VLPs particles with an average diameter of ~ 17 nm were observed **(S1B Fig)**. Consistently, dynamic light scattering (DLS) results show that nearly 99% of the capsid proteins self-assemble into particles in solution with an average radius of 8.36 nm **(S1C Fig)**. Next, we investigated whether or not the NLS sequence is important for VLP assembly in addition to its accessory role in the replication of PCV. Instead of replacing the NLS sequence by a non-NLS sequence, we completely deleted the N-terminal 1–45 residues and replaced them with a fused N-terminal His-tag “MGSSHHHHHHSSGLVPRGSH” derived from pET-28b vector to make PCV2-His-ΔN45. As expected, no stable PCV2 particles were observed in solution, as confirmed by TEM, DLS and analytical FPLC.

### Crystal and cryo-EM structures of PCV2 VLP

To explore the structural roles of N-terminal fragments of PCV2 capsid protein in PCV2 assembly, multiple PCV2 constructs were used for crystallization screening and structure determination. Among all the constructs screened, PCV2-His-ΔN45 was able to be concentrated to ~10 mg/ml and was successfully crystallized and optimized. Single-wavelength high-resolution diffraction data was collected in Taiwan synchrotron radiation resource center (NSRRC) and scaled to a space group of P2_1_, with the cell parameters as a = 194.12Å, b = 201.88Å, c = 231.28Å and β = 90.72° at the resolutions of 2.8Å. The structure was determined by MORDA/CCP4 by using 3R0R as the search model [29], and the model was refined by REFMAC/CCP4 and rebuilt using Coot. There was one icosahedral particle per asymmetric unit with crystallographic statistics listed in **Table 1**.

**Table 1:** Data collection and refinement statistics.

**Data collection**	**PCV2 VLP**
Space group	P2_1_
PDBID	5ZJU
Wavelength (Å)	1
Cell dimensions	
*a (Å)*	194.12
*b (Å)*	201.88
*c (Å)*	231.28
*β (°)*	90.72
Molecule/ASU	60 subunits
Resolution (Å) ^a^	2.80 (2.85–2.80)
R_sym_ (%) ^a^	9.6 (42.6)
*I/*σ*(I)*	13.5 (2.4)
Completeness (%) ^a^	99.4 (99.1)
Redundancy ^a^	3.8 (3.9)
**Refinement**	
Search Model	3R0R
Resolution (Å) ^a^	2.80 (2.88–2.80)
No. reflections	410,467
R_work_ (R_free_) (%)	16.3/22.4 (24.6/31.9)
No. atoms	
Protein	92,840
Water	4,846
B-factors (Å^2^)	
Protein	43.11
Water	32.21
R.m.s. deviations	
Bond lengths (Å)	0.018
Bond angles (°)	1.678
% favored (allowed) in Ramachandran plot	91.6 (8.4)

^a^ Values for the highest-resolution shell are in parentheses.

**Fig 1:**
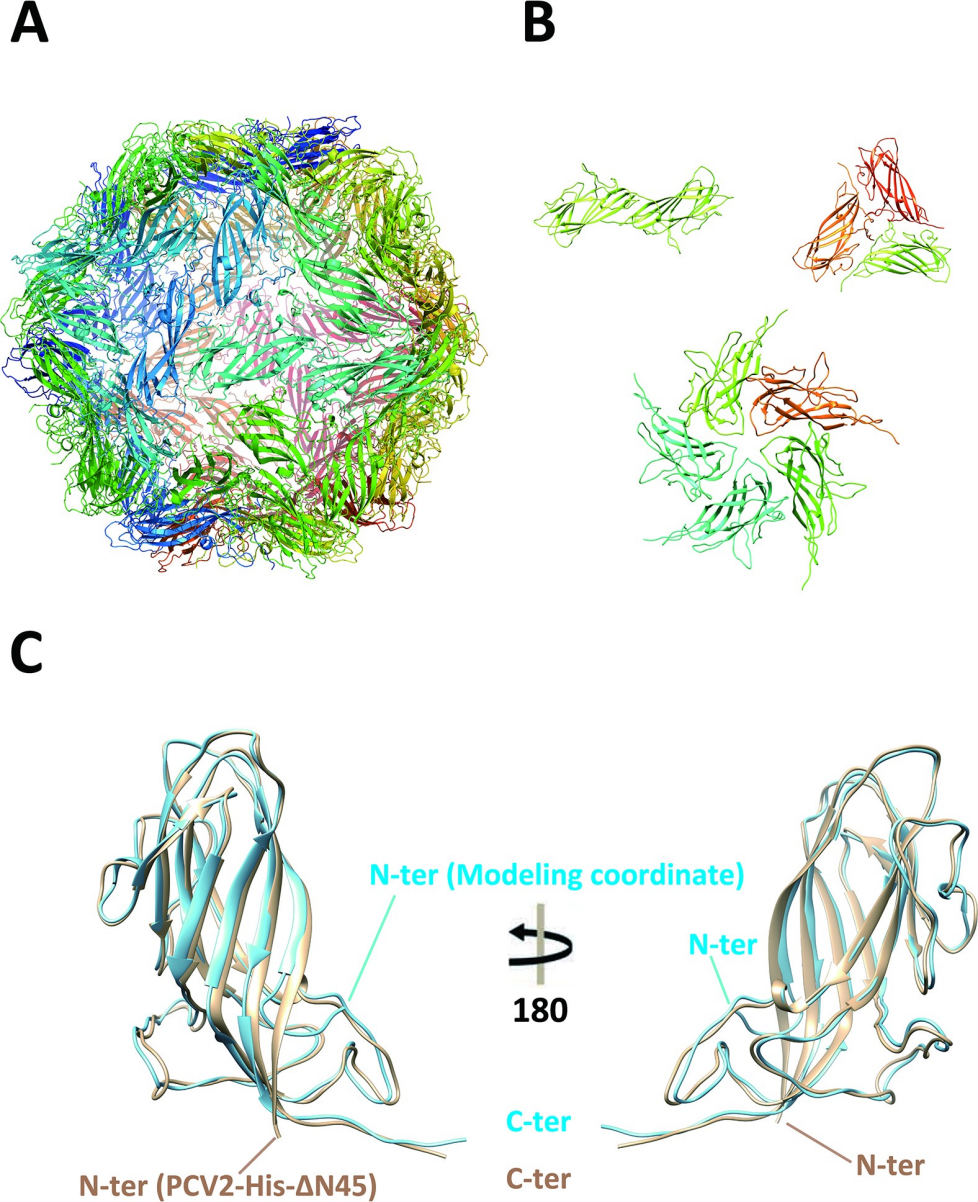
Crystal structure of PCV2-His-ΔN45 VLPs. **(A)** Ribbon representation of crystal structure of PCV2-His-ΔN45 VLP at 2.8Å assembled from 60 PCV2 capsid protein subunits. **(B)** The association of subunits in dimer, trimer and pentamer form, presented from VLPs. **(C)** Structural comparison of crystal structure of PCV2-His-ΔN45 subunit and cryo-EM structure of full-length PCV2 subunit. The crystal structure of PCV2-His-ΔN45 subunit is colored in light yellow, whereas the cryo-EM structure of full-length PCV2 subunit is colored in light blue.

Surprisingly, although PCV2-His-ΔN45 does not self-assemble into particle simultaneously in solution at relatively low concentration (no more than 2mg/ml), the crystal structure of PCV2-His-ΔN45 showed that PCV2-His-ΔN45 capsid proteins indeed self-assemble into VLP within the crystals. At such conditions, the crystal structure of PCV2-His-ΔN45 displayed an icosahedral VLP structure consisting of 60 capsid protein subunits **(Fig 1A)** with clearly observable 2-fold, 3-fold and 5-fold axes **(Fig 1B, S2A and S2B Fig)**. In our crystal structure, the interpretable densities for the PCV2-His-ΔN45 start at residue ^43^Ser **(Fig 1C)**, and the densities for most of the surface loops, such as loop-BC, loop-CD and loop-EF etc, are clearly visible and easily traced **(Fig 1A)**. Although the extra 20 non-authentic residues “MGSSHHHHHHSSGLVPRGSH” were introduced to the vector expressing PCV2-His-ΔN45 protein, the traceable non-authentic sequences are only two residues long ^43^SH^44^, which are located within the interior region of the PCV2 VLP. The extremely flexible nature of N-terminal fragment of PCV2 capsid protein strongly suggests that neither the NLS sequence nor the particular positively charged residues are absolutely essential for PCV2 icosahedral symmetry packing and/or particle assembly under this particular crystallization condition.

**Fig 2:**
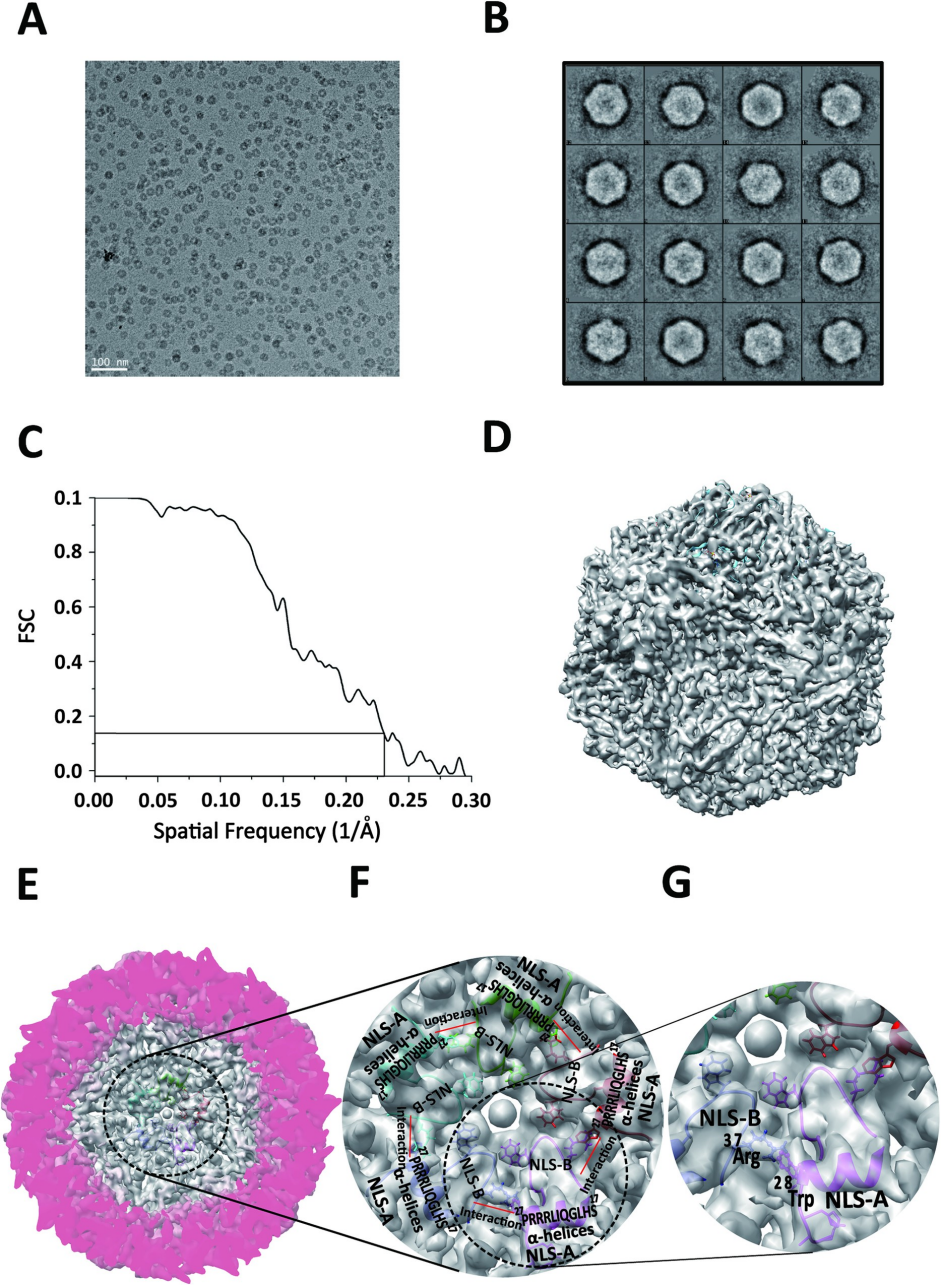
Cryo-electron microscopy structure of full-length PCV2 VLPs. **(A)** Typical raw cryo EM image of PCV2 VLPs imaged at the magnification of 67,000× under Titan Krios electron microscopy. **(B)** 2D classification of PCV2 VLP particles from the EM images collected under 300 kV. **(C)** The Fourier Shell Correlation curve of resolution for our current reconstruction is shown. The line corresponds to the gold standard criterion for resolution estimation (FSC 0.143). **(D)** The 3D reconstructed cryo-EM structure of PCV2 VLP reveals a typical T = 1 icosahedral particle fold. **(E)** The closest half of the density map removed to reveal internal structural feature of the NLS fragment. **(F)** Cryo-EM density map surrounding NLS region. The density maps for NLS-B, α-helices etc are indicated. **(G)** Cryo-EM density map surrounding NLS region. The cation-π interaction between residues ^28^Trp and ^37^Arg is labeled.

To further validate the structural features observed from the crystal structure determined and to investigate whether or not *in vitro* assembled PCV2-VLPs indeed resemble the intact PCV2 viruses, more than 150 cryo-EM micrographs of *in vitro* assembled full-length PCV2-VLPs were collected under low dose condition. Approximately 12,000 individual PCV2 particles were excised and boxed from the selected 92 best micrographs. Next, contrast transfer function (CTF) correction for each micrograph was determined individually and the Fourier transformation was applied to each image by EMAN2 (blake.bcm.tmc.edu/EMAN2) **(Fig 2A)**. Thirty-two groups of particles with different reflection orientations were built and averaged. Among them, only the good average classes with an apparent icosahedral shape were used to make the initial model **(Fig 2B)**. Roughly 9,600 particles were used for 3D reconstruction, which reveals a typical PCV2 viral capsid structure at 4.12Å resolution **(Fig 2C)** with an icosahedral T = 1 symmetry comprising 60 capsid protein subunits **(Fig 2D, S2C and S2D Fig)**. The densities corresponding to PCV2 capsid proteins were extracted from the reconstructed density map and viewed by UCSF Chimera software (www.cgl.ucsf.edu/chimera/‎) [32,33]. The local resolution distribution was assessed by Resmap/Chimera [34] **(S3A–S3C Fig)**, showing significant resolution variation ranging from 5~6.0Å on the outer surface and inner concave regions to 3.5~4.0Å at β-strand barrel regions **(S3B Fig)**. The relatively low resolutions on the outer surface and inner concave regions of the VLP capsid suggest the structural flexibility of PCV2 capsid at these regions **(S3B Fig)**. Most of the main chains and some of the side chains of PCV2 capsid protein were clearly traced and the initial model was further refined by twenty rounds of real-space group refinement using PHENIX [35] **(S3D Fig)**.

In our cryo-EM structure, the density of each capsid subunit is clearly visible even at r.m.s.d = 4.1 Å, showing a typical circovirus capsid protein fold consisting of eight β-strands joined by several loops located on the surface of the particle. By contrast, some surface loops, such as BC-loop (^58^KRTTVKTPS^66^) and CD-loop (^79^FLPPGGGSNPRSVPEE^94^), are relatively difficult to trace and build due to the weak densities, suggesting the structural flexibility of these exposed surface loops. The N-terminal fragments, such as fragments ^15^PRSHLGQILRRRP^27^ and ^33^RHRYRWRRKN^42^ surrounding the N-terminal NLS region, are clearly visible on our cryo-EM density map contoured at level of 3.6 e/Å^3^ (r.m.s.d = 3.60 Å.), suggesting the relative structural rigidity of these N-terminal fragments **(Fig 2E and 2F)**. In our cryo-EM structure, Arginine-rich residues (^15^PRSHLGQILRRRP^27^) next to NLS-A reach into the adjacent capsid protein located within PCV2 VLP chamber, and interact with its NLS-B fragment (^33^RHRYRWRRKNG^43^) to stabilize the VLP formation **(Fig 2F and 2G)**. Moreover, the charges and types of these amino acids could also play certain roles in determining the rate and/or stability for VLP assembly, probably due to the interior interactions of the amino acids proximal to the 5-fold axis. There is no density map available for N-terminal fragment ^1^MTYPRRRYRRRRHR^14^ due to structural flexibility at this region, strongly suggesting that this N-terminal fragment may not be essential for icosahedral capsid formation and stability. Table 2 shows the statistics of the refined PCV2 VLP model derived from our cryo-EM density map.

**Table 2:** Data collection and refinement of cryo-EM structure of full-length PCV2 VLP.

**Data Collection**	
Voltage (kV)	300
Dose (e^-^/Å^2^)	25
Detector	Falcon II
Pixel size (Å)	1.69
Defocus range (μm)	-1.0 ~ - 4.0
**Reconstruction (RELION)**	
Micrographs (Initial/ Final)	151/92
Particle number (Initial)	12,000
Particle number (Final)	9,600
Symmetry	icosahedral
Box size (pixels)	200
Accuracy of rotations	0.927°
Accuracy of translations (pixels)	0.508
Sharpening B-factor (Å^2^)	-140.93
Final resolution (Å)	4.12
EMDB accession code	EMD-6746
**Model Refinement (PHENIX)**	
Cross correlation (Whole Volume)	0.791
Cross correlation (Masked)	0.781
Ramachandran Plot	
Outliers	0.61%
Allowed	12.12%
Favored	87.27%
**PDB accession code**	5ZBO^a^

^a^ Due to the low resolution and structural flexibility, only the coordinates of a.a 45–231 of full-length PCV2 VLP are included in PDB-5ZBO.

In literatures, the non-traceable N-terminal residues are presumed to be located proximal to the icosahedral 5-fold axes. However, no clear structure has been modeled due to the structural flexibility of N-terminal part of the determined cryo-EM structure [29,30]. Remarkably, in our crystal structure of PCV2-His-ΔN45 at 2.8Å resolution, the traceable fragment ^43^GIFNTRL^49^ (the numerical is based on the authentic sequence NO.) is located along the 1^st^ β-strand toward the interior core of the VLP. By contrast, in the cryo-EM structure, the traceable fragment ^43^SHFNTRL^49^ (PCV2-full-length) forms a loop structure pointing to the exterior direction with a β-turn formed by ^47^Thr and ^48^Arg **(Fig 1C)**. This observation suggests that the N-terminal fragment might be exposed at the capsid surface to mediate the virus-host interaction by “breathing”, which is often observed by other virus [36–38].

Though the NLS was truncated, the icosahedral VLPs derived from PCV2-His-ΔN45 capsid protein were observed within the crystal structure, probably due to crystal packing force in crystallization buffer. By contrast, PCV2-His-ΔN45 capsid protein itself is not able to self-assemble into stable VLP in solution no matter in low or high concentration **(S4A–S4D Fig)**. We speculate that the N-terminal fragment, including the NLS motif, play an important role in VLP stabilization. Consistently, the cryo-EM structure of non-tagged full-length PCV2 VLP at 4.12 Å resolution clearly shows that the interpretable N-terminal α-helix fragment ^15^PRSHLGQILRRRP^27^ interacts with the adjacent NLS-B fragment ^33^RHRYRWRRKNG^43^ located in the interior concavity, which is involved in VLP stabilization **(Fig 2F and 2G)**.

**Fig 3:**
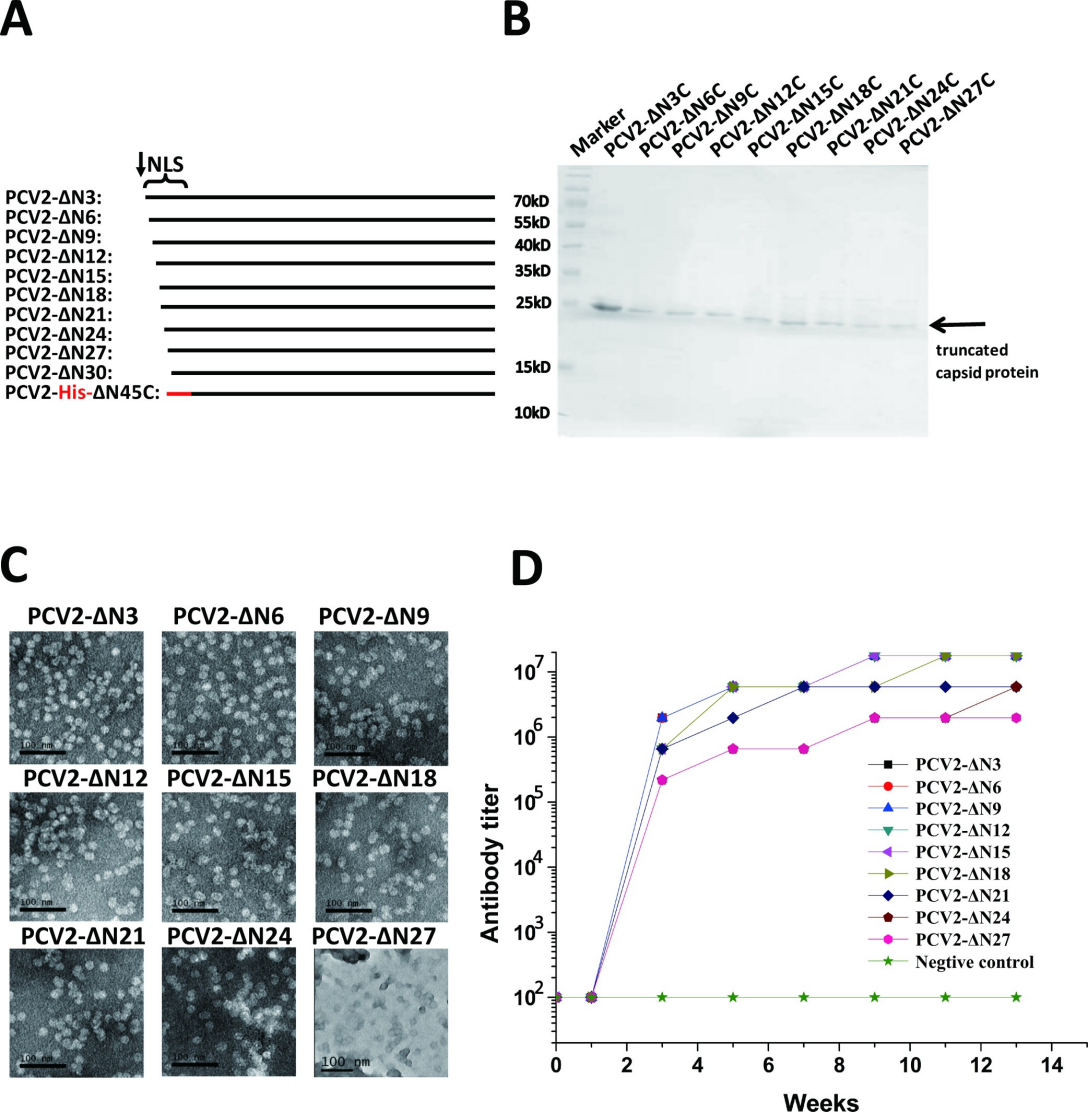
Characterization of PCV2 VLPs derived from *in vitro* expressed and assembled full-length and truncated PCV2 capsid proteins. **(A)** Schematic drawing of full-length and truncated PCV2 capsid proteins. The NLS region was indicated as brace and detailed sequence are shown in S6 Fig. **(B)** SDS-PAGE gel of purified PCV2 truncated capsid proteins (PCV2-ΔN3, PCV2-ΔN6, PCV2-ΔN9, PCV2-ΔN12, PCV2-ΔN15, PCV2-ΔN18, PCV2-ΔN21, PCV2-ΔN24, and PCV2-ΔN27) with the PCV2 monomers indicated with arrows. **(C)** Transmission electron microscopy of VLPs assembled from purified truncated PCV2 capsid proteins (PCV2-ΔN3, PCV2-ΔN6, PCV2-ΔN9, PCV2-ΔN12, PCV2-ΔN15, PCV2-ΔN18, PCV2-ΔN21, PCV2-ΔN24, and PCV2-ΔN27). **(D)** The mouse poly-antibody titers against PCV2 VLPs assembled from truncated PCV2 capsid proteins (PCV2-ΔN3, PCV2-ΔN6, PCV2-ΔN9, PCV2-ΔN12, PCV2-ΔN15, PCV2-ΔN18, PCV2-ΔN21, PCV2-ΔN24, and PCV2-ΔN27).

To further investigate the structural roles of N-terminus of PCV2 capsid protein in PCV2 VLP assembly, we have systematically expressed and purified a series of truncated non-tagged PCV2 capsid proteins by truncation of every 3 residues, up to 30 residues **(Fig 3A and 3B)**, from N-terminus to test VLP assembly ability by both dynamic light scattering (DLS) and transmission electron microscopy (TEM) assays. As expected, DLS results show that PCV2 capsid proteins (from full-length to ΔN27) self-assemble into particles with an average radius of ~ 8.5 nm in solution **(S5 Fig)**. Consistently, these PCV2 particles imaged under transmission electron microscopy show that the diameter (2 × radius) of the majority of the PCV2 VLP particles is ~17 nm in the solution **(Fig 3C)**. By contrast, PCV2 capsid protein with N-terminal 30 residues truncation (PCV2-ΔN30) was not able to form icosahedral VLP, which is confirmed by DLS, size exclusion chromatography (SEC) and TEM methods **(S6 Fig, Table 3)**.

**Table 3:** DLS, SEC and EM of PCV2 truncated proteins.

	Dynamic light scattering(DLS)			SEC Retained	TEM size
	2×Rh	%Poly	%Intensity	volume (ml)	(nm)
	(nm)	diversity			
PCV2-ΔN3	16.7	12.1	96.5	10.11	~ 17
PCV2-ΔN6	16.7	21.4	96.7	10.13	~ 17
PCV2-ΔN9	16.9	15.7	87.9	10.15	~ 17
PCV2-ΔN12	17.3	14.9	93.2	10.27	~ 17
PCV2-ΔN15	16.6	24.6	92.5	10.24	~ 17
PCV2-ΔN18	16.71	21.5	96.7	10.19	~ 17
PCV2-ΔN21	16.78	20.83	100	10.11	~ 17
PCV2-ΔN24	16.59	18.9	95.1	10.18	~ 17
PCV2-ΔN27	16.45	12.4	92.3	10.16	~ 17
PCV2-ΔN30	3.22	15.7	87	17.9	NA
PCV2-His-Δ45	2.5	22.5	81	17.6	NA

Since the *in vitro* assembled N-terminal truncated PCV2-VLPs (no more than 27 residues) and full-length PCV2 VLPs have similar icosahedral shape and diameters, immune assays were performed to check the immunogenicity for these VLPs. Purified and *in vitro* assembled VLPs supplemented with adjuvant were injected into mouse, and poly-antibody mouse serum were collected every week for 12 weeks after immunization. Then enzyme-linked-immune-sorbent assays (ELISA) were performed to measure the antibody titers. As expected, antibody titers increased rapidly after the second booster vaccination and most of the antibody titers reached 10^6^−10^8^ **(Fig 3D)**. As compared to the full-length PCV2-VLPs, most of the truncated PCV2-VLPs (PCV2-ΔN3 to PCV2-ΔN27) triggered similar immunogenicity responses from mice **(Fig 3D)**.

### Mapping PCV2 type-specific neutralizing epitope

Porcine circovirus contains several genotypes, such as PCV1 and PCV2. Notably, although the sequences of these two genotypes are quite similar, the phenotypes vary significantly. PCV2 is considered a threatening pathogen, causing PMWS in piglets, whereas PCV1 is not. However, more and more data shows that the PCV viruses keep evolving under selection pressure and more infectious strains and genotypes could evolve by genome recombination of spreading PCV viruses with some genotypes that may not cause immediate PMWS associated symptoms among the infected piglets. Hence, it is critical to develop novel diagnostic methods to monitor the co-spreading of different PCV strains and genotypes among farms.

**Fig 4:**
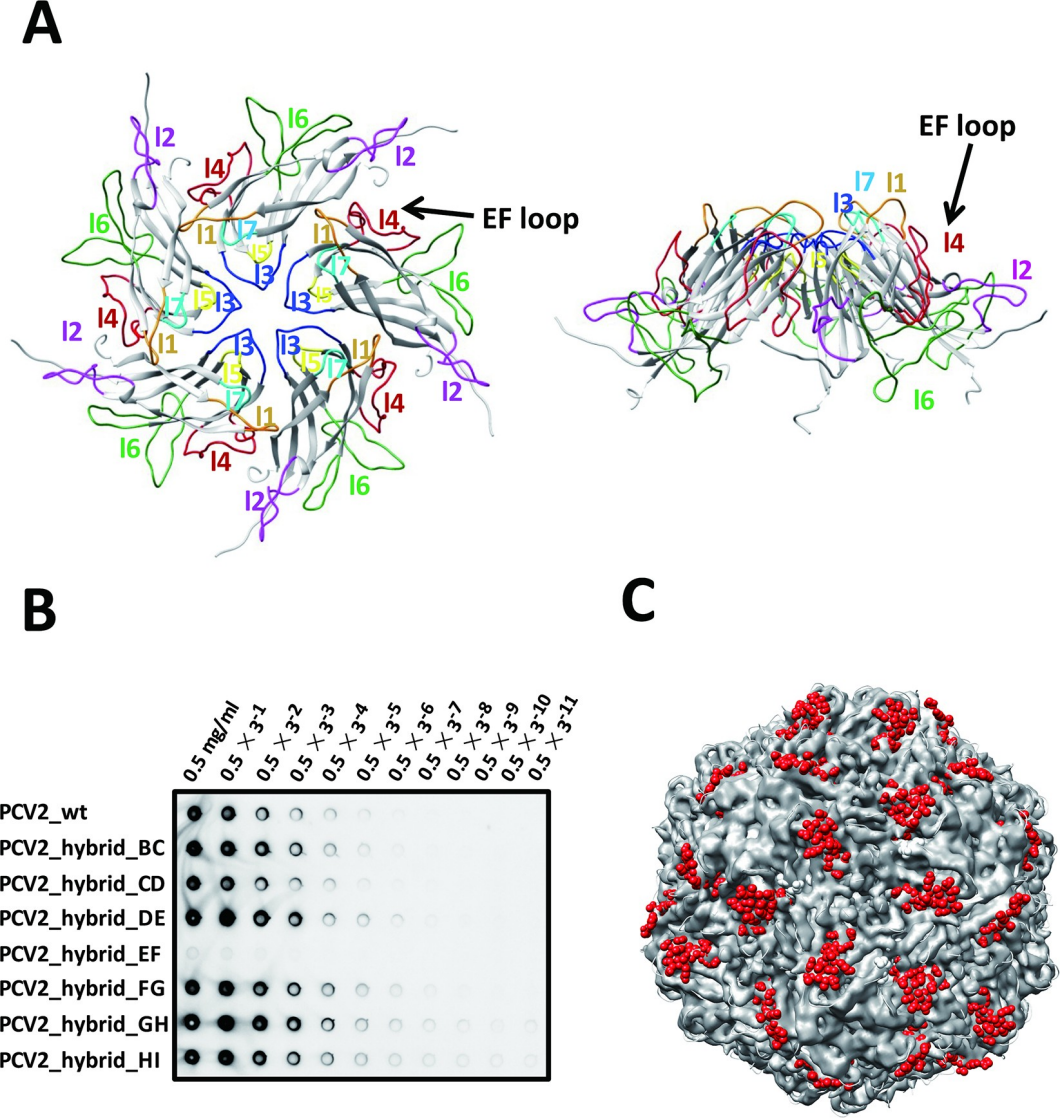
PCV2 type-specific neutralizing epitope mapping. **(A)** Ribbon diagram of the PCV2 pentamer structure viewed from the side and top orientations, respectively. The seven flexible surface loops of PCV2 pentamer are indicated. **(B)** Bio-dot western blot detection of affinities between PCV2 type-specific mAb and chimeric PCV2 capsid proteins with swapped loops from PCV1. Swapping of PCV2 capsid protein EF-loop with the corresponding PCV1 capsid protein loop disrupts the binding between PCV2 capsid protein and 3H11 mAb. **(C)** Cryo-EM density map of full-length PCV2 VLP. The PCV2 type-specific EF-loops are colored in red, whereas the rest of the PCV2 VLP densities are colored in grey.

Sequence alignment of the capsid protein sequences of several currently spreading PCV1 and PCV2 strains showed that the majority of the deviated sequences are located within the surface loop regions **(S7 Fig)**. Hence, we speculate that PCV2 type-specific mAb could have the ability to distinguish PCV2 from PCV1 by recognizing one of the surface epitopes. Previous results showed that the capsid sequence changes among the analyzed circovirus isolates do not yield any major structural changes in the viral capsid assembly but instead to the antigenic regions [39]. Hence, we assume that loop replacement will only change the local structures at the epitope regions but not the overall β-strands core region and capsid viral assembly. To this end, we performed systematic affinity screening between a PCV2 type-specific neutralizing mAb, named 3H11 [40], and chimeric PCV2 capsid by replacing the capsid protein surface loop sequence by corresponding PCV1 capsid protein sequence. We have made seven chimeric PCV2 capsid proteins by replacing the loop sequences at the following loop regions one by one: BC-loop (residues 58–66), CD-loop (residues 79–94), DE-loop (residues 108–116), EF-loop (residues 124–146), FG-loop (residues 153–156), GH-loop (residues 162–193) and HI-loop (residues 204–208) **(S1 Table)**. Bio-dot ELISA assays were used to test the binding specificities between the PCV2 type-specific 3H11 mAb and the purified wild-type/chimeric proteins. As expected, 3H11 mAb specifically recognizes PCV2 EF-loop (residues 128–143) since the replacement of PCV2 EF-loop sequence by corresponding PCV1 EF-loop sequence prevented the binding of 3H11 to PCV2 **(Fig 4A and 4B)**. In contrast, replacement of other PCV2 surface loops had marginal impact on 3H11 binding with purified chimeric PCV2 capsid proteins. Sequence comparison also suggested that residues 134–139 of EF-loop could be responsible for 3H11 binding due to large sequence differences between PCV1 and PCV2 capsid proteins **(S7 Fig)**. Consistently, both high-resolution X-ray and cryo-EM structures showed that these residues are stretched out from the capsid surface and exposed for mAb binding **(Fig 4C)**.

**Fig 5:**
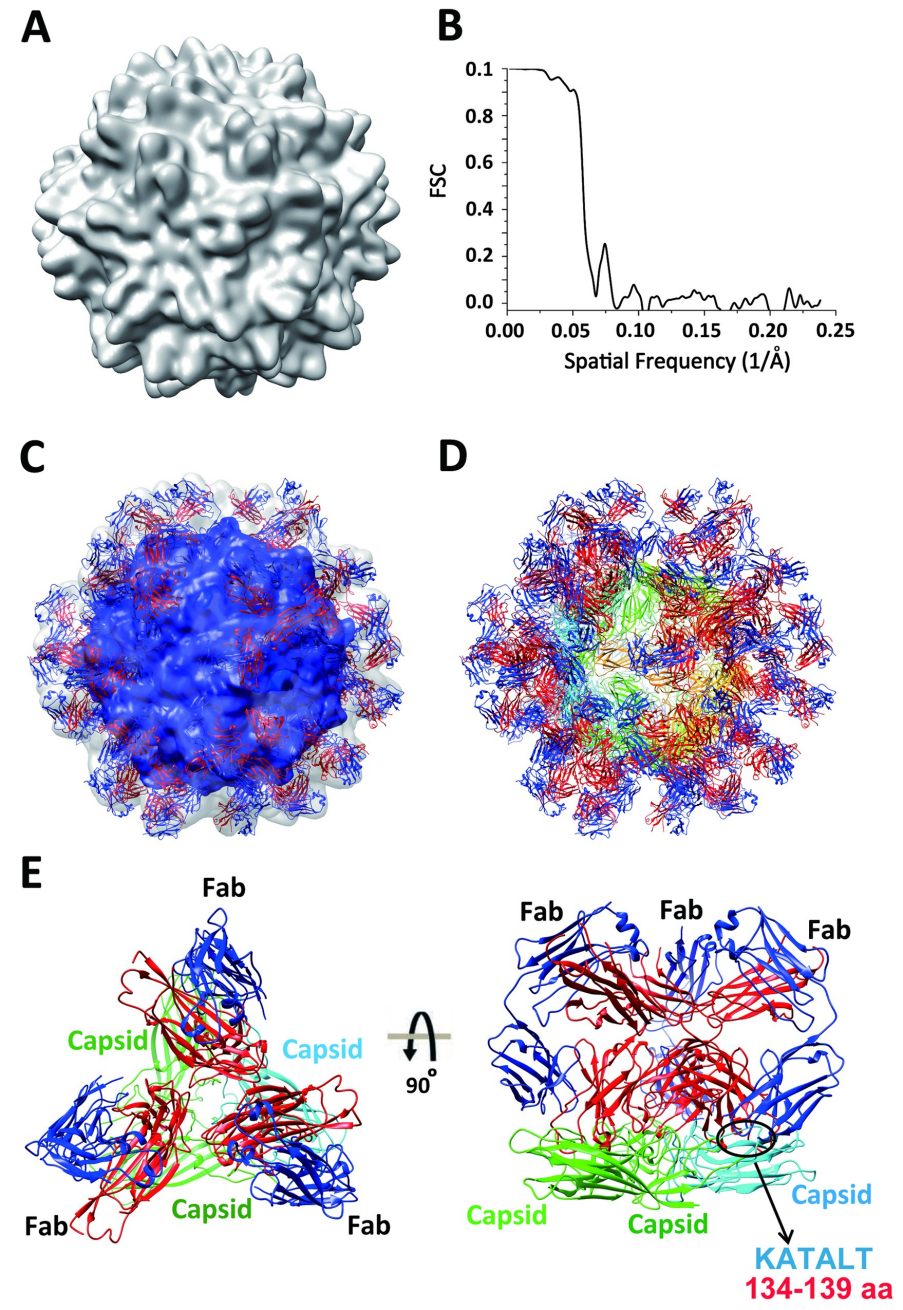
The cryo-EM structure of PCV2 VLP in complex with 3H11 Fab. **(A)** The 3D reconstructed cryo-EM structure of PCV2 VLP in complex with 3H11-Fab reveals a typical T = 1 icosahedral particle fold. **(B)** The Fourier Shell Correlation curve of resolution for cryo-EM structure is shown. The line corresponds to the gold standard criterion for resolution estimation (FSC 0.143). **(C)** Segmentation and of structural model of Fab fragments binding with PCV2 VLP. The crystal structure of PCV2 VLP (PCV2-His-ΔN45) and the homology model structure of mAb-3H11-Fab were fitted into the densities of the 3D-reconstructed complex. The density maps of outer and inner shell are shown as 60% and 10% transparent surfaces, respectively, and the fitted atomic models are represented by ribbons. The densities corresponding to PCV2 VLP and 3H11 Fab fragments are colored in blue and grey, respectively. **(D)** Structural models of PCV2 VLP and mAb-3H11. **(E)** The atomic model of PCV2 VLP is colored by subunit and labeled accordingly. The heavy chain and light chain of mAb-3H11 Fab fragment are colored in red and blue, respectively. This structure clearly shows that 3H11 Fab binds to EF-loop located on the PCV2 VLP surface.

To further investigate the structural detail of the PCV2 type-specific neutralizing epitope, we have determined the cryo-EM structure of PCV2 VLP in complex with 3H11-Fab fragment. Roughly 21,280 particles were used for 2D classification, and only good classes were selected for model building and refinement. The cryo-EM structure of the complex revealed an icosahedral symmetry with two-layer architecture comprising 60 copies of PCV2-3H11-Fab (**Fig 5A**). The densities of PCV2 VLP and Fab fragments were segmented and extracted from the density map and visualized using UCSF Chimera software package (www.cgl.ucsf.edu/chimera/), which revealed the clear densities assigned to the Fab fragment of 3H11 and PCV2 VLP particles. In this structure, determined to ~15 Å when the 0.143 Fourier correlation criterions were used **(Fig 5B)**, the inner shell is composed of 60 copies of PCV2 and the outer shell is composed of 60 copies of 3H11-Fab fragments. To understand the molecular details of PCV2 type-specific epitope recognition by 3H11, we docked the crystal structure of our PCV2 VLP and the homology model structure of 3H11 Fab, derived from a Rosetta calculation, into our cryo-EM density map of the complex one by one **(Fig 5C and 5D)**. At ~15 Å resolution, by taking advantage of the PCV2 VLP structure determined at high resolution and the conserved Fab structure, we were able to dock both PCV2 and 3H11 Fab models into the cryo-EM density of PCV2 in complex with 3H11-Fab (~4% atoms are outside at current contour level). In our docked model, the CDR regions of 3H11 Fab clearly bind to the protruding EF-loop located on the PCV2 VLP surface, further confirming that PCV2 EF-loop (residues 128–143) contains a PCV2 type-specific neutralizing epitope **(Fig 5E, S8 Fig)**. Taken together, these data demonstrated that 3H11 precisely recognizes the PCV2 type-specific neutralizing epitope located on the surface EF-loop region.

## Discussion

PCV2 intensifies the spreading of severe porcine syndromes worldwide, causing immuno-suppression and co-morbidity with other dangerous pathogens, leading to severe economic loss in the swine industry. Commercially available PCV2 vaccines, including classical vaccines derived from inactivated PCV2 viruses and recombinant vaccine, have proven to be efficient in preventing the spread of PCV2. In previous studies, different expression systems were used to express and assemble PCV2 VLP from PCV2 capsid protein. Among them, the *E*. *coli* expression system was considered the ideal manufacturing system due to its low cost and robust scalability. However, limited high-resolution structural data were available to fully capture the structure and immunogenicity of *in vitro* expressed and assembled PCV2 VLP derived from *E*. *coli* system.

### Structural roles of PCV2 capsid protein N-terminus in PCV2 VLP assembly

The PCV2 capsid protein is the only protein responsible for PCV2 capsid formation and the dominant immunogenic host response. Our structural prediction suggests that PCV2 N-terminal fragment up to 40 a.a. could be structurally flexible, which is partially confirmed by the N-terminal truncated PCV2 structures published in literature [29,30] **(S9A–S9D Fig)**. The full-length PCV2 capsid protein was considered very difficult to express in soluble form in numerous protein expression systems [41], suggesting the N-terminal fragment of PCV2 capsid protein could play a role in protein folding and/or capsid formation [42]. Consistent with these findings, N-terminal fragment truncated PCV2 capsid protein showed compromised immunogenicity and disability in PCV2 capsid assembly [43]. Notably, N-terminal fragment of PCV2 capsid protein, including several batches of Arginine-rich positively charged residues and a predicted α-helix embedded within the NLS sequences, is highly conserved among PCV family members, suggesting N-terminal fragment of PCV2 capsid protein does have functional roles in PCV2 particle assembly and/or PCV2 replication **(S10 Fig)**. However, due to the difficulty for full-length PCV2 capsid protein expression, no solid structural information of this N-terminal fragment available to illuminate the roles of these basic residues in capsid assembly and/or PCV2 replication at both molecular and structural level.

In this study, we have reported a robust *E*. *coli* expression system expressing non-tagged full-length PCV2 capsid proteins, a series of N-terminal truncated PCV2 capsid proteins and one His-tagged N-terminal truncated PCV2 capsid protein in soluble form: PCV2-FL, PCV2-ΔN3, PCV2-ΔN6, PCV2-ΔN9, PCV2-ΔN12, PCV2-ΔN15, PCV2-ΔN18, PCV2-ΔN21, PCV2-ΔN24, PCV2-ΔN27, PCV2-ΔN30 and PCV2-His-ΔN45. Notably, both non-tagged PCV2-FL and all non-tagged PCV2 capsid proteins that were truncated up to 27 residues at the N-terminal are able to form regular icosahedral VLP with almost identical morphology and immunogenicity. The His-tagged NLS deleted capsid protein, PCV2-His-ΔN45, forms icosahedral VLP only with the assistance of crystal packing since PCV2-His-ΔN45 itself is not able to form stable VLP at both low and high concentrations. Remarkably, in our cryo-EM structure determined at 4.12Å, clear densities are observed for the authentic N-terminal fragment ^15^PRSHLGQILRRRP^27^ including an α-helix, which interacts with the enriched Arginine-rich NLS-B (^33^RHRYRWRRKNG^43^) fragment located proximal to the β-barrel to stabilize the VLP formation in solution.

Hence, based on these structural results, we speculate that PCV2 capsid protein could self-assemble in solution into PCV2 VLP without the assistance of N-terminal sequence. However, such VLP derived from N-terminal truncated PCV2 capsid protein, lacking the interactions between the α-helix in NLS from one capsid protein and NLS-B fragment from an adjacent capsid protein is instable and easily disassembled. Therefore, PCV2 N-terminus of PCV2 capsid protein, including the NLS fragment, play pivotal roles in PCV2 VLP stabilization rather than PCV2 VLP formation.

### Heparin binding site

Viral invasion often involves interactions between virus surface proteins and host receptors and surface molecules, such as glycosaminoglycans (GAGs), heparin sulfate and other carbohydrate molecules. Although detailed *in vivo* mechanisms could be significantly different from those observed in *in-vitro* assays, the heparin sulfate binding motif located at the surface of the PCV2 capsid has been proposed to play a critical role in viral invasion. In literature, biochemical analysis suggests that the heparin sulfate binding motif is located at PCV2 D-β strand, whereas structural analysis suggests that the binding motif is located at PCV2 DE-loop [44,45].

Our high resolution structures, determined by both X-ray crystallography (crystal packing) and cryo-EM (in solution) approaches, provide a unique chance for us to re-examine the potential heparin binding sites at the PCV2 capsid surface. As expected, several patches of basic residues (XBBXBX or XBBBXXBX) are observed at the PCV2 capsid surface. Among them, a putative heparin binding motif (^98^IRKVKV^103^) was revealed from sequence analysis. However, this binding motif is located in the interior of the capsid VLP and significant structural rearrangement should be triggered upon host receptor binding if this motif is indeed involved in receptor binding. However, this kind of structural flexibility was not observed in the study of our PCV2 structures, as determined by X-ray crystallography and cryo-electron microscopy. Another conserved positively charged patch (^179^KRNQLWLR^186^) was observed on GH-loop, which is located proximal to the icosahedral 3- and 5-fold axes. However, this residue patch is quite flexible and may not be the heparin binding site, as the dramatic structural re-arrangement at the 3- and 5-fold axes could disrupt the capsid formation.

At the other hand, PCV2 contains a lengthy Arginine-rich motif at its N-terminus. The NLS sequence is divided into NLS-A (^5^RRRYRRRRHRPR^16^) and NLS-B (^33^RHRYRWRRK^41^) by one α-helix (^15^RRRYRRRRHRPR^24^) **(S10 Fig)**. Although the structure of NLS-A fragment was not revealed in our cryo-EM analysis, the structure of the predicted α-helix (^15^PRSHLGQILR^24^) adjacent to NLS-A and the structure of NLS-B (^33^RHRYRWRRK^41^) were clearly traced and built **(Fig 2F)**. We speculate that the flexible NLS fragments including the α-helix ^15^PRSHLGQILR^24^ could be the heparin binding sites because: 1) NLS residues are located at the virus’ surface with flexible basic-charged patches which could interact with host receptors and other host surface molecules; 2) NLS residues are located far away from the capsid assembly axes so that structural rearrangement of the VLP does not disrupt PCV2 capsid formation; 3) the well-refined NLS (^15^PRSHLGQILR^24^) fragment is indeed an α-helix, which meets the prediction that heparin binding sites should be located near a rigid α-helix or β-strand; 4) PCV2 NLS fragment is used as a cell-penetrating peptide to enhance intracellular delivery of plasmid DNA during viral infection [46,47]. Therefore, our research, partially supported by our cryo-EM structure, strongly suggests that the flexible PCV2 N-terminal NLS fragment located at the PCV2 capsid surface could serve as the heparin binding site involved in the viral infection on host cells.

### PCV2 type-specific neutralizing epitope

In literature, several PCV2 type-specific epitopes were predicted based on structural and biochemical analyses. High-resolution structures of PCV2 VLP suggested that some of the seven surface loops may serve as the epitopes eliciting PCV2 type-specific antibodies [29,48]. However, the biochemical and structural data differ in confirming these epitopes.

To identify and validate the PCV2 type-specific neutralizing epitopes, which are crucial for PCV2 vaccine and diagnosis kits development, we have systematically screened for a PCV2 type-specific neutralizing monoclonal antibody and mapped the recognition sites back to the surface of PCV2 VLP. We were able to isolate one neutralizing mAb which specifically recognizes PCV2 instead of PCV1. Follow-up PCV1/PCV2 loop swapping experiments, followed by bio-dot ELISA experiments, showed that this particular mAb recognizes PCV2 EF-loop (^134^KATALT^139^), which is an exposed loop located at the PCV2 capsid surface revealed by both X-ray and cryo-EM structures.

Notably, although PCV1 and PCV2 infection are widely spread in swine farms, postweaning multisystemic wasting syndrome is caused primarily by PCV2. In practice, PCV2 capsid proteins have been widely used as a diagnostic antigen for serologic detection of PCV2 infection. However, PCV1 was frequently misdiagnosed as PCV2 due to the high sequence similarity between PCV1 and PCV2 capsid protein. In this study, based on the 15Å cryo-EM structure of PCV2 VLP in complex with 3H11-Fab, we were able to, for the first time, identify that EF-loop (residues ^134^KATALT^139^) is indeed a PCV2 type-specific neutralizing epitope. The full-length of PCV2 EF-loop contains ~23 residues, in which residues 127–138 are entirely exposed to the surface and highly divergent between PCV1 and PCV2. ^134^KATALT^139^ is the most divergent fragment within the EF-loop. Hence, the identification that EF-loop is the PCV2 type-specific neutralizing epitope should provide a new strategy for the next generation PCV2 vaccine design and generation of PCV2 type-specific antigens for PCV2 diagnosis.

### PCV2 vaccine selection pressure

The global genotypic shift from PCV2a to PCV2b occurred around 2003 [49]. It was followed by another shift from the predominant PCV2b to PCV2d in more recent times [20–23]. The newly emerged PCV2d, identified in PCV2 vaccine failure investigations, might have the ability to replicate in pigs under vaccination pressure [21]. Due to the emergence of a new PCV2 genotype, concerns have been raised over the efficacy of current commercial PCV2 vaccines [50]. Although commercially available PCV2a-based vaccines have been shown to have cross-protection against PCV2d (mPCV2b) in a PRRSV-mPCV2b-challenge mode [50], field trials suggest that the efficacy of these PCV2 vaccination may be PCV2 genotype-dependent [51]. Therefore, it is urgent to develop a broad spectrum PCV vaccine to prevent another wave of newly emerged PCV from spreading worldwide.

In literature, commonly developed classes of broad-spectrum vaccines are: broad-spectrum vaccines, polyvalent vaccines and structure-based vaccines [52]. In broad-spectrum vaccines, antigens with broad spectrum neutralizing epitopes are capable of conferring protection against a range of viral genotypes. Polyvalent vaccines are synthesized through a mixture of antigens from several of a virus’ genotypes into a single vaccine and have been shown to be as effective as targeted monovalent vaccines [53]; for viruses with a limited number of genotypes, such as PCV2 (PCV2a ~ PCV2d), it is practical to develop a conjugated polyvalent vaccine from existing prevalent genotypes. Structure-based vaccines, containing multiple epitopes (both B- and T-cell epitopes), elicit neutralizing antibody responses to several genotypes of a virus [54]. Structure-based vaccines could be designed using the latest proteomic techniques and bioinformatics tools, which rely on type-specific neutralizing epitope identification.

In this manuscript, our results detail these two points: 1) Structural roles of PCV2 capsid protein N-terminus in PCV2 particle assembly. 2) The identification of a PCV2 type-specific neutralizing epitope. We identified PCV2b-specific neutralizing epitope, located on the EF-loop (^134^KATALT^139^). Based on the sequence homology, this EF-loop region is conserved in PCV2a and PCV2b as ^134^KATALT^139^, and conserved in PCV2c and PCV2d as ^134^KANALT^139^. Therefore, it is possible to design a broad-spectrum prophylactic vaccine that targets both PCV2a+2b and PCV2c+2d, effectively overcoming vaccine selection pressure. The PCV2 type-specific neutralizing epitope identified in this work is not only able to differentiate between non-pathogenic PCV1 and PCV2 –a necessity in PCV diagnostic kits—, but can also be used for PCV structure-based design of a chimera vaccine.

In summary, by the combination of X-ray crystallography and cryo-EM approaches, we successfully observed the structural principles of PCV2 assembly, PCV2 type-specific neutralizing epitopes and PCV2 heparin binding sites. Our efforts not only revealed the structural details of *E*. *coli* expressed PCV2 VLPs, providing an efficient approach to manufacture low-cost high efficient VLP vaccines against PCV2 infection; but also identified the PCV2 type-specific neutralizing EF-loop epitope, providing a new strategy to design new diagnosis kits effectively discriminating threatening PCV2 from non-threatening PCV1 strains.

## Materials and methods

### Ethics statement

The protocol of animal study was approved by the Committee on the Ethics of Animal Experiments of the National Research Center for Veterinary Medicine (Permit Number: 20160313088). The study was conducted following the Guide for the Care and Use of Animals in Research of the People's Republic of China.

### Cloning, protein expression and purification

Open reading frame 2 (ORF2) of porcine circovirus type 2 (PCV2) was amplified by DNA polymerase and sub-cloned into a modified pET vector to express the full-length capsid protein without tag. The constructed expression plasmid pET-ORF2 was transformed into *Escherichia coli* BL21 (DE3) competent cell in presence of Kanamycin antibiotics. The cells were grown in LB media supplemented with Kanamycin antibiotics to an OD_600_ value reaching 0.6, a final concentration of 0.4 mM IPTG was added to induce the recombinant protein expression. After cultured overnight, cells were harvested with a centrifuge and re-suspended in lysis buffer containing 20 mM Tris (pH 7.4), 500 mM NaCl, 1 mM DTT and 2 mM EDTA. The cells suspension was kept on ice and lysed using a homogenizer for 4 times. After ultra-centrifuge by 40,000 rpm for 1 hour, the supernatant was collected and purified by ion exchange chromatography and hydrophobic chromatography columns.

The truncated (PCV2-ΔN3 to PCV2-ΔN30) capsid proteins were expressed and purified using the similar strategies by PCR amplifying the corresponding DNA sequences from the full-length PCV2 capsid protein gene and sub-cloned into the similar pET vector without N-terminal His-tag. The N-terminal truncated and His-tagged PCV2 (PCV2-His-ΔN45) gene was amplified from the corresponding DNA sequences from the full-length PCV2 capsid protein gene and sub-cloned into pET28a vector with N-terminal. The PCV2-His-ΔN45 protein was purified by Histidine affinity chromatography column, followed by ion exchange chromatography and hydrophobic chromatography columns. The loop swapped chimeric PCV2 clones were generated by swapping the corresponding PCV1 capsid sequences (^58^KGGYSQPS^66^, ^79^FLPPSGGTNPLPLPEQ^94^, ^108^RDPITSNER^116^, ^124^ILDANFVTPSTNLAYDPYINYSS^146^, ^153^PFTY^156^, ^162^TPKPELDKTIDWFHPNNKRNQLWLHLNTHTNV^193^, ^204^NAATA^208^) to the PCV2 capsid protein sequence, respectively, followed by the same protein expression and purification protocols described. All the clones were validated by sequencing.

### Size-Exclusion chromatography

The capsid proteins (full-length and truncated proteins) were purified at 4°C by using an ÄKTA automatic protein purification system (GE Healthcare ÄKTA explorer 10 system). Before sample loading, the prepackaged Supdex200 column was equilibrated with the buffer containing 20 mM sodium phosphate (pH 6.5) and 500 mM NaCl. After centrifugation for 10 mins, the supernatant of the purified sample was loaded onto High Performance gel filtration columns (GE Healthcare) at the flow rate of 0.5 ml/min. After loading, the eluted sample was collected by a 96 wells fraction collector, and checked by 12% acrylamide SDS-PAGE gel. The retention volume of each run of the full-length or truncated capsid proteins was recorded, and all the curves are fitted together to compare the size of the samples.

### PCV2 antibody generation and characterization

After mixed with Freund’s complete adjuvant, recombinant full-length and truncated capsid proteins were injected into BALB/c mice, respectively. Same amount of BSA alone mixed with Freund’s complete adjuvant was injected in mice as negative control. A booster immunization with the same dosage of PCV2 capsid protein mixed with Freund’s incomplete adjuvant was administered 2 weeks later. The serums of these immunization mice were collected every week, and the anti-PCV2 antibody titers were measured by indirect ELISA. Followed the routine hybridoma protocol, the spleen cells were collected from immunized mouse and fused with SP2/0 myeloma cells. Hybridomas were obtained by limiting dilution, and the antibody generated was checked by indirect ELISA. After selected, the cultured hybridoma cells were injected into pristane-treated BALB/c mice to gain ascetic fluids, in which the antibody was purified by Protein-A affinity chromatography.

### Indirect Enzyme-linked immunosorbent assay (ELISA)

Purified truncated PCV2 capsid proteins (PCV2-ΔN3, PCV2-ΔN6, PCV2-ΔN9, PCV2-ΔN12, PCV2-ΔN15, PCV2-ΔN18, PCV2-ΔN21, PCV2-ΔN24, PCV2-ΔN27) were coated on a 96-well ELISA plate with stepwise decrements amount in a buffer containing 20 mM phosphate buffer (pH 6.5), 500 mM NaCl. After blocked by blocking buffer, the serum was added and the mixture was incubated at 37°C for 30 mins. After 3 times washing, followed by the addition of secondary IgG-HRP (Goat against mice), the titer measurement was performed according to the instructions of Light Shift Chemiluminescent EMSA kit (Thermo Fisher Scientific Inc., USA). The antibody titers in the serum were recorded and calculated by the reading score multiple by dilution times.

### Bio-dot blotting

Our cryo-EM structure of complex of PCV2 VLP and mAb-3H11 Fab fragments showed that the PCV2 type-specific epitope is located within the EF-loop of PCV2 capsid protein. To validate the structural work, bio-dot blotting assay were performed to detect binding between mAb-3H11 and PCV2/PCV1 loop swapping hybrid capsid proteins. Purified wide-type and PCV2/PCV1 hybrid capsid proteins (PCV2-WT, PCV2-hybrid-BC, PCV2-hybrid-CD, PCV2-hybrid-DE, PCV2-hybrid-EF, PCV2-hybrid-FG, PCV2-hybrid-GH, and PCV2-hybrid-HI) were coated on a Nitrocellulose membrane by 96-well Bio-dot device (Bio-Rad) with stepwise decrements amount in a buffer containing 20 mM phosphate buffer (pH 6.5), 500 mM NaCl. After blocked by blocking buffer, mAb-3H11 was added and the mixture was incubated at 37°C for 30 min. After washing for 3 times, secondary IgG-HRP (Goat again mice) was added and the detection was performed according to instruction of Light Shift Chemiluminescent EMSA kit (Thermo Fisher Scientific Inc., USA). The image was collected on the G-Box biomolecular imager (GE Healthcare).

### Dynamic light scattering assay

The DLS measurements were performed at room temperature on a DynaPro (protein solution) DLS instrument. Before measuring, all protein samples and control buffers were firstly filtered through a 0.22μm filter to avoid any dust and unwanted aggregates, and degassed on a thermal vacuum, followed by ultracentrifuge for at least 15 mins. The measurement cuvette was rinsed with Milli-Q water, 100% methanol and filtered water again for several times to cleanse and remove dust. The sample buffer, served as a blank, was firstly measured and protein samples were measured thereafter. For each sample, at least 20 acquisitions were collected for data analysis.

### Crystallization and structure determination of PCV2 VLP

PCV2-His-ΔN45 capsid protein was concentrated to ~12mg/ml and screened for crystallization conditions by hanging drop method. More than 500 conditions were screened and the best quality crystals were obtained in the buffer containing 2 M ammonium sulfate, 0.1 M citrate acid (pH4.6). After optimization, single crystals were picked and flash frozen in a cryo-protection solution containing the crystallization buffer plus 15% glycerol. Single wavelength (at 1.0 Å) diffraction data was collected at Taiwan National Synchrotron Radiation Resource Center (NSRRC) and processed using HKL2000 software [55]. The structure was determined by MORDA/CCP4 (www.ccp4.ac.uk) using 3R0R as the search model [29]. The model was built using the program Coot and refined to 2.8Å resolution using REFMAC/CCP4. The crystallographic statistic detail of the structure is listed in **Table 1**. The structural and electrostatics figures were prepared using PyMOL (Delano Scientific).

### Negative stain electron microscopy (EM) of N-terminal truncated PCV2 VLP

5 μl purified N-terminal truncated PCV2 VLPs specimens (PCV2-ΔN3, PCV2-ΔN6, PCV2-ΔN9, PCV2-ΔN12, PCV2-ΔN15, PCV2-ΔN18, PCV2-ΔN21, PCV2-ΔN24, PCV2-ΔN27 and PCV2-ΔN30) were applied to carbon coated copper grids, respectively. The grids were treated by phosphato-tungstic acid (PTA) and dried. Images were taken on a FEI Tecnai 12 electron microscope operated at 120 kV, with a magnification of 67,000×.

### Cryo-EM image collection and structure determination of full-length PCV2 VLP and PCV2 VLP in complex with 3H11-Fab

5 μl purified PCV2 VLPs specimen and PCV2 VLP in complex with 3H11-Fab fragment were loaded onto a carbon coated Quatifoil 2/1 grid, respectively, blotted with filter paper (two times, each time per 2 seconds) and rapidly plunged into liquid ethane, pre-cooled by liquid nitrogen. Cryo-EM images were taken from the frozen grids in a FEI Titan Krios TEM cryo electron microscope operated at 300 kV, with a magnification of 67, 000× and a pixel size of 1.69Å/pixel. Measured defocus values of these images range from -1 μm to -4 μm. The details were shown in the cryo-EM data processing flowchart for the full-length PCV2 VLPs **(S11 Fig)**.

### Accession numbers

Protein Data Bank: The structure factor and coordinate for PCV2-His-ΔN45 have been deposited with accession codes 5ZJU. EM data bank: The cryo-EM structures of full-length PCV2 VLP and PCV2 VLP in complex with 3H11-Fab have been deposited to EMDB with the accession codes EMD-6746 and EMD-6961, respectively. The model coordinate of full-length PCV2 VLP has been deposited to EMDB with the accession codes 5ZBO.

## Supporting information

S1 Table Details of loops region of PCV1 and PCV2 capsid protein. (PDF)

S1 Fig Full-length PCV2 capsid proteins assemble into VLPs. **(A)** SDS-PAGE gel of purified PCV2 capsid protein with the dimer and monomer are indicated. **(B)** Transmission electron microscopy of PCV2 VLPs. The scale-bar is 100 nm long and EM result indicated that the PCV2 capsid proteins are assembled into VLPs. **(C)** Dynamic light scattering measurement of PCV2 VLPs. The enlarged area showed that the average hydrodynamic radius of PCV2 VLP is 8.36 nm and the re-assembly rate is 98.5%.
(PDF)

S2 Fig Structural comparison of PCV2 VLPs determined by either cryo-EM or X-ray crystallography approaches. (A) Density map of crystal structure of PCV2-His-ΔN45 VLP. (B) Structural model of PCV2-His-ΔN45 VLP. (C) Cryo-EM density map of full-length PCV2 VLP. (D) Refined structural model of full-length PCV2 VLP.
(PDF)

S3 Fig Local resolution assessment of reconstructed 3D cryo-EM structure of full-length PCV2 VLPs. (A) PCV2 VLP slices through input volume. (B) PCV2 VLP slices through ResMap results. (C) Histogram of PCV2 VLP ResMap results. (D) Refined cryo-EM structural model of full-length PCV2 VLP reconstructed at 4.12Å. The structural details of PCV2 at the surface loop regions are enlarged and shown.
(PDF)

S4 Fig Transmission electron microscopy of PCV2-His-ΔN45. (A) TEM of PCV2-His-ΔN45 (1.25 mg/ml). (B) TEM of PCV2-His-ΔN45 (2.5 mg/ml). (C) TEM of PCV2-His-ΔN45 (5 mg/ml). (D) TEM of PCV2-His-ΔN45 (10 mg/ml).
(PDF)

S5 Fig Dynamic light scattering measurement of truncated PCV2 capsid proteins in VLP assembly. (PDF)

S6 Fig HPSEC of truncated PCV2 capsid proteins in VLP assembly. (PDF)

S7 Fig Multiple sequence alignment of full-length PCV1 and PCV2 capsid proteins. Sequence alignment of 5 strains of full-length PCV2 capsid proteins with 5 strains of full-length PCV1 capsid proteins by ClustalW. The secondary structure is shown above the aligned sequences, α-helices are displayed as helices, and seven loops are also indicated.
(PDF)

S8 Fig Plot Range of Modeling coordinates of cryo-EM structure of PCV2 VLP+ 3H11 Fab complex by RIVEM. (PDF)

S9 Fig Comparison with other PCV2 capsid protein structures. **(A)** Ribbon diagram of the PCV2 monomer model (PDBID: 3JCI) derived from EM structure at 2.9Å (EMD-6555). **(B)** Ribbon diagram of the PCV2 monomer model derived from the crystal structure of PCV2-His-ΔN45 at 4.12Å (PDBID: 5ZJU). **(C)** Ribbon diagram of the PCV2 monomer model derived from crystal structure of PCV2-N12 at 2.3Å (PDBID: 3R0R). **(D)** Structural comparison of PCV2 monomers from different resources.
(PDF)

S10 Fig Multiple sequence alignment of full-length PCV2 capsid sequences. Sequence alignment of 61 PCV2 capsid sequences using ClustalW. The number and positive charged residues, including Arg and Lys, are labeled, and seven surface loops are also indicated.
(PDF)

S11 Fig A flow-chart for the cryo-EM data processing and 3D reconstruction of the full-length PCV2 VLP using Relion software (version 1.4). (PDF)
