# Supplementary material for: Structural roles of PCV2 capsid protein N-terminus in PCV2 particle assembly and identification of PCV2 type-specific neutralizing epitope
Source: PLoS Pathog. 2019 Mar 1;15(3):e1007562. doi: 10.1371/journal.ppat.1007562 (PMC6415871; doi:10.1371/journal.ppat.1007562)
Supplement: S1 Table — (PDF) [file ppat.1007562.s001.pdf]

S1 Table. Mo et al.

S1 Table. Details of loops region of PCV1 and PCV2 capsid protein

		PCV1		PCV2	
		Residues	Aligned sequence	Residues	Aligned sequence
L1:	BC loop	60-68	KGGYSQPS	58-66	KRTTVKTPS
L2:	CD loop	81-96	FLPPSGGTNPLPLPEQ	79-94	FLPPGGGSNPRSVPEE
L3:	DE loop	110-118	RDPITSNER	108-116	CSPITQGDR
L4:	EF loop	126-148	ILDANFVTPSTNLAYDPYINY SS	124-146	ILDDNFVTKATALTYDPYVNY SS
L5:	FG loop	155-158	PFTY	153-156	PFSY
L6:	GH loop	164-195	TPKPELDKTIDWFHPNNKRN QLWLHLNTHTNV	162-193	TPKPVLDSTIDYFQPNNKRN QLWLRLQTAGNV
L7:	HI loop	206-210	NAATA	204-208	NSIYD
