# Supplementary figures and images for: Structural roles of PCV2 capsid protein N-terminus in PCV2 particle assembly and identification of PCV2 type-specific neutralizing epitope
Source: PLoS Pathog. 2019 Mar 1;15(3):e1007562. doi: 10.1371/journal.ppat.1007562 (PMC6415871; doi:10.1371/journal.ppat.1007562)

S1 Fig. Mo et al.

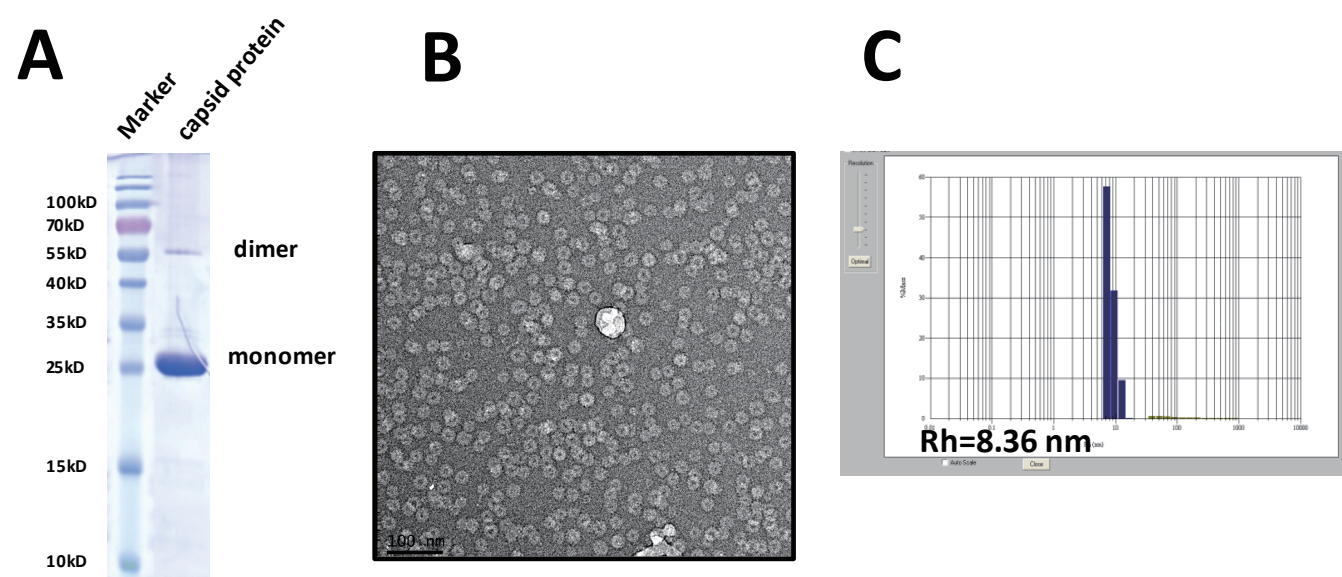

Full-length PCV2 capsid proteins assemble into VLPs.

Supplement: S1 Fig — (A) SDS-PAGE gel of purified PCV2 capsid protein with the dimer and monomer are indicated. (B) Transmission electron microscopy of PCV2 VLPs. The scale-bar is 100 nm long and EM result indicated that the PCV2 capsid proteins are assembled into VLPs. (C) Dynamic light scattering measurement of PCV2 VLPs. The enlarged area showed that the average hydrodynamic radius of PCV2 VLP is 8.36 nm and the re-assembly rate is 98.5%. (PDF) [file ppat.1007562.s002.pdf]

**S4 Fig. Mo et al.**

**A**

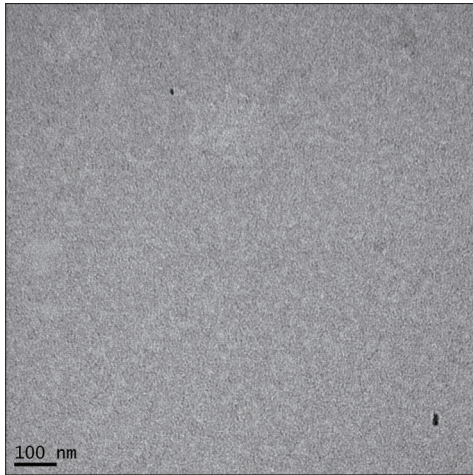

**B**

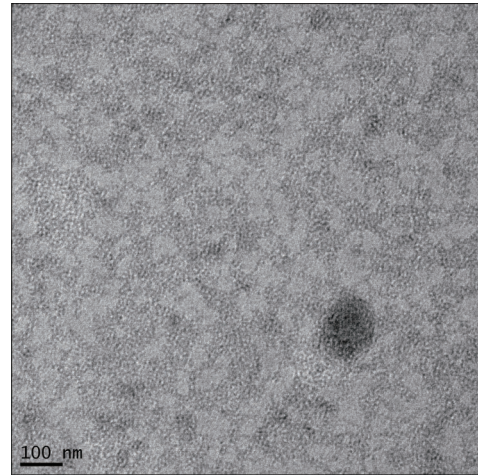

**C**

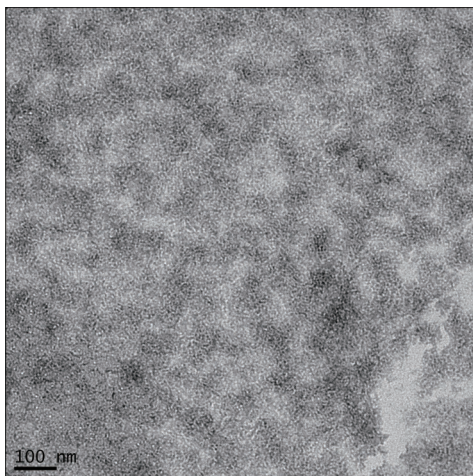

**D**

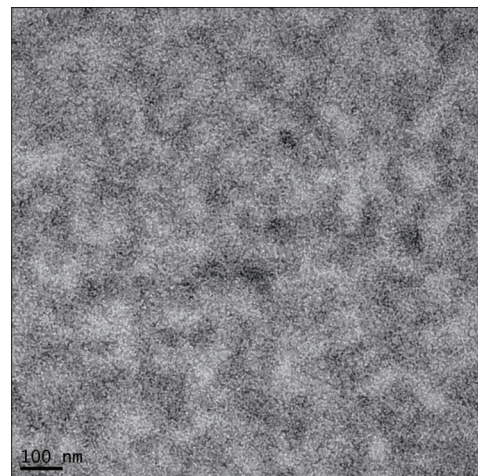

**Transmission electron microscopy of PCV2-His- $\Delta$ N45**

Supplement: S4 Fig — (A) TEM of PCV2-His-ΔN45 (1.25 mg/ml). (B) TEM of PCV2-His-ΔN45 (2.5 mg/ml). (C) TEM of PCV2-His-ΔN45 (5 mg/ml). (D) TEM of PCV2-His-ΔN45 (10 mg/ml). (PDF) [file ppat.1007562.s005.pdf]

S6 Fig . Mo et al.

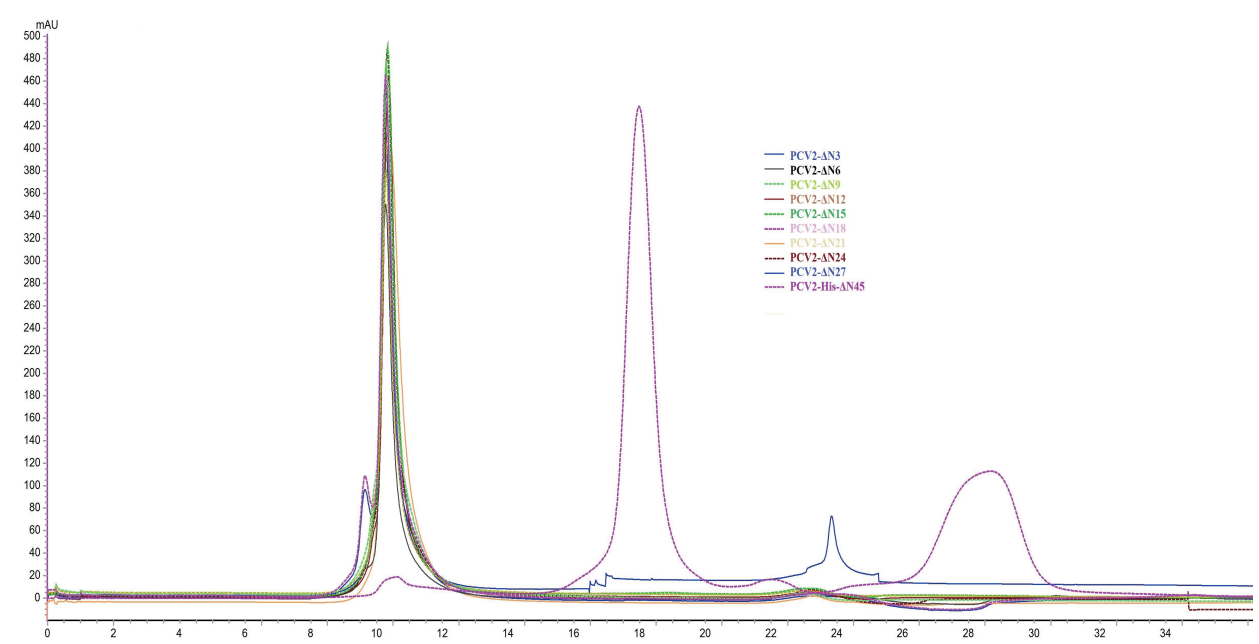

HPSEC of truncated PCV2 capsid proteins in VLP assembly

Supplement: S6 Fig — (PDF) [file ppat.1007562.s007.pdf]

S7 Fig. Mo et al.

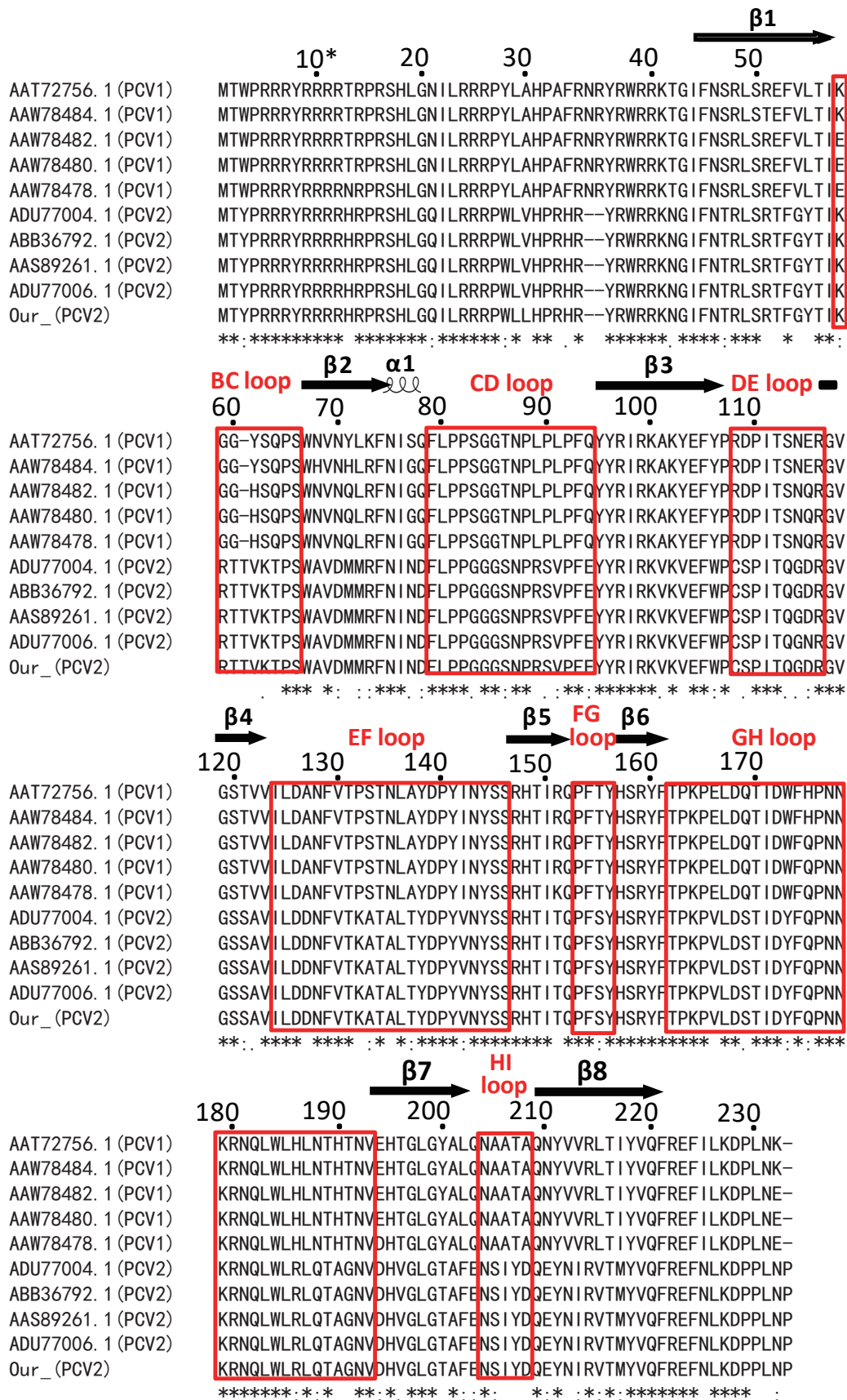

Supplement: S7 Fig — Sequence alignment of 5 strains of full-length PCV2 capsid proteins with 5 strains of full-length PCV1 capsid proteins by ClustalW. The secondary structure is shown above the aligned sequences, α-helices are displayed as helices, and seven loops are also indicated. (PDF) [file ppat.1007562.s008.pdf]

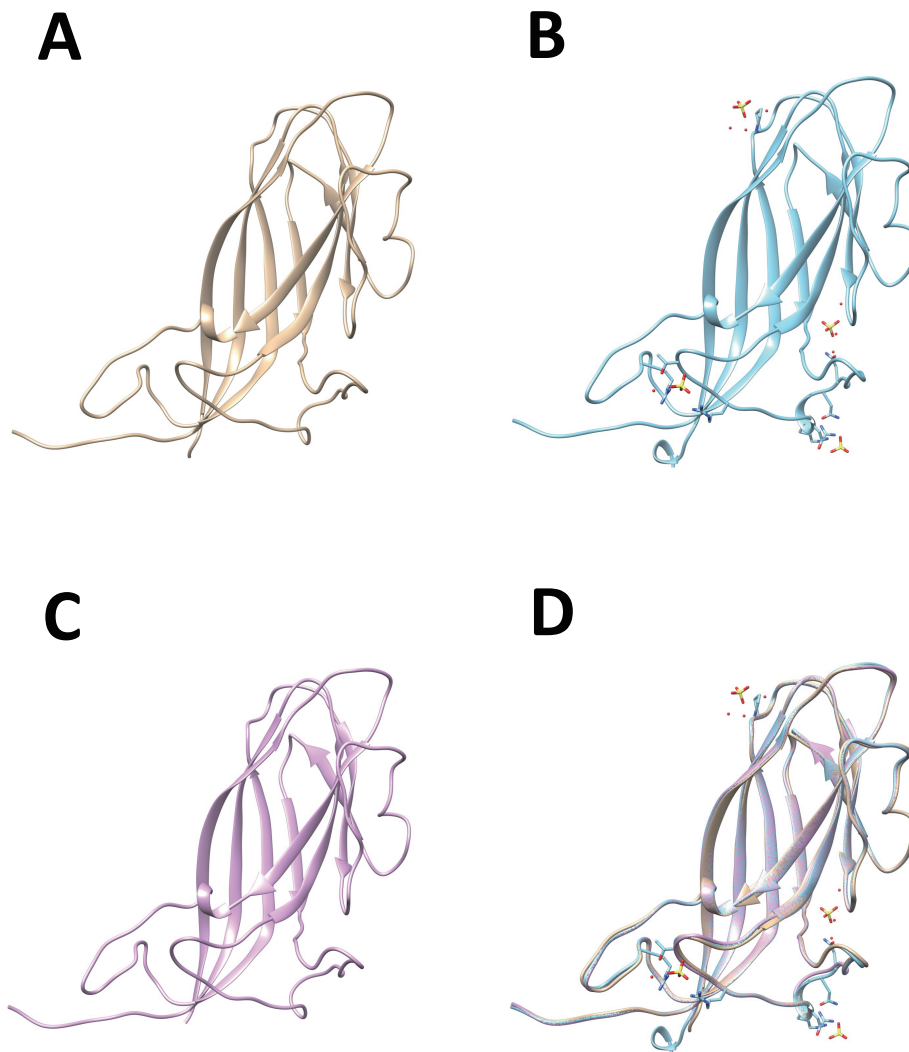

Comparison with other PCV2 capsid protein structures

Supplement: S9 Fig — (A) Ribbon diagram of the PCV2 monomer model (PDBID: 3JCI) derived from EM structure at 2.9Å (EMD-6555). (B) Ribbon diagram of the PCV2 monomer model derived from the crystal structure of PCV2-His-ΔN45 at 4.12Å (PDBID: 5ZJU). (C) Ribbon diagram of the PCV2 monomer model derived from crystal structure of PCV2-N12 at 2.3Å (PDBID: 3R0R). (D) Structural comparison of PCV2 monomers from different resources. (PDF) [file ppat.1007562.s010.pdf]

S10 Fig. Mo et al.

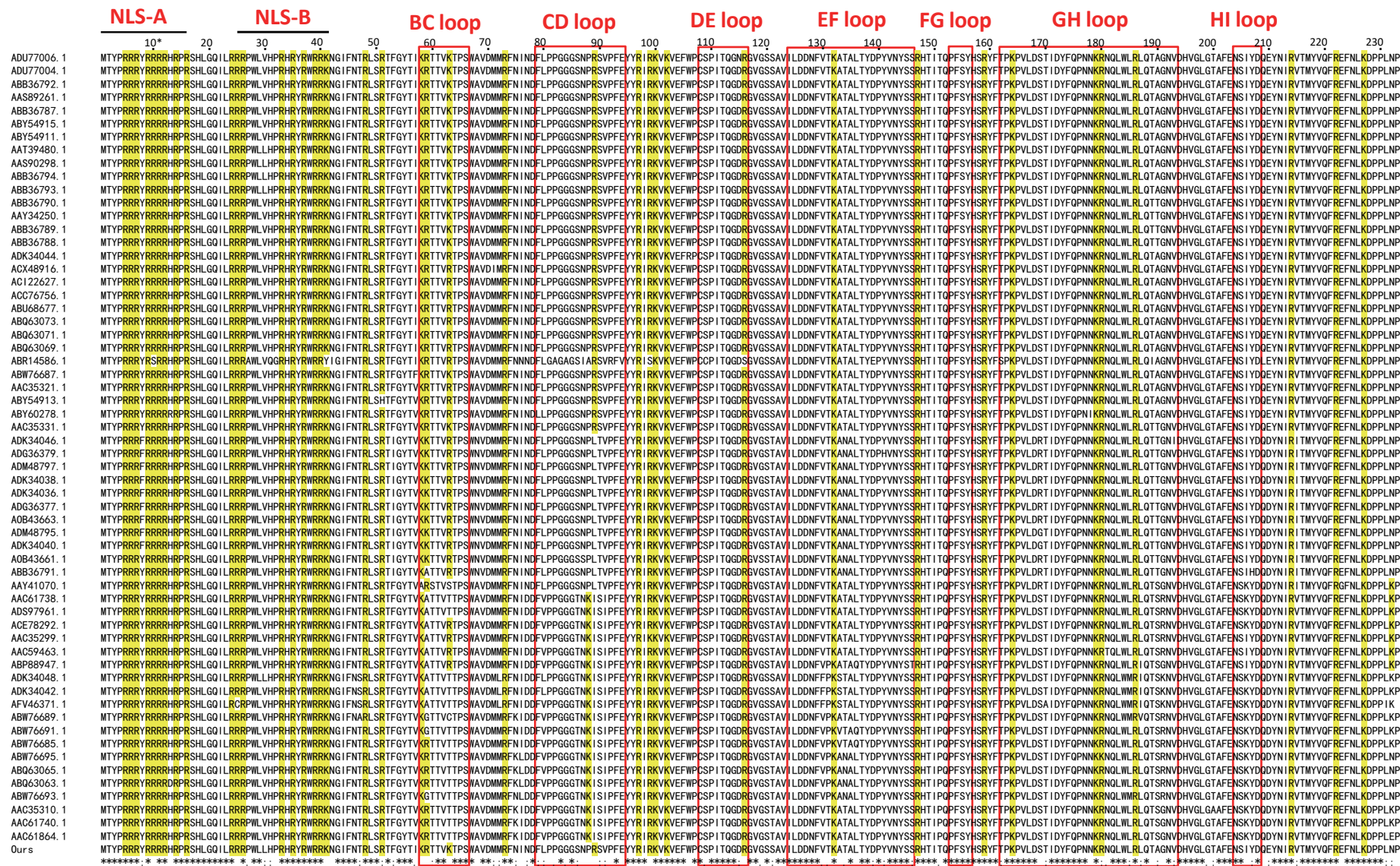

Supplement: S10 Fig — Sequence alignment of 61 PCV2 capsid sequences using ClustalW. The number and positive charged residues, including Arg and Lys, are labeled, and seven surface loops are also indicated. (PDF) [file ppat.1007562.s011.pdf]
