# Supplementary material for: Structural roles of PCV2 capsid protein N-terminus in PCV2 particle assembly and identification of PCV2 type-specific neutralizing epitope
Source: PLoS Pathog. 2019 Mar 1;15(3):e1007562. doi: 10.1371/journal.ppat.1007562 (PMC6415871; doi:10.1371/journal.ppat.1007562)
Supplement: S2 Fig — (A) Density map of crystal structure of PCV2-His-ΔN45 VLP. (B) Structural model of PCV2-His-ΔN45 VLP. (C) Cryo-EM density map of full-length PCV2 VLP. (D) Refined structural model of full-length PCV2 VLP. (PDF) [file ppat.1007562.s003.pdf]

S2 Fig. Mo et al.

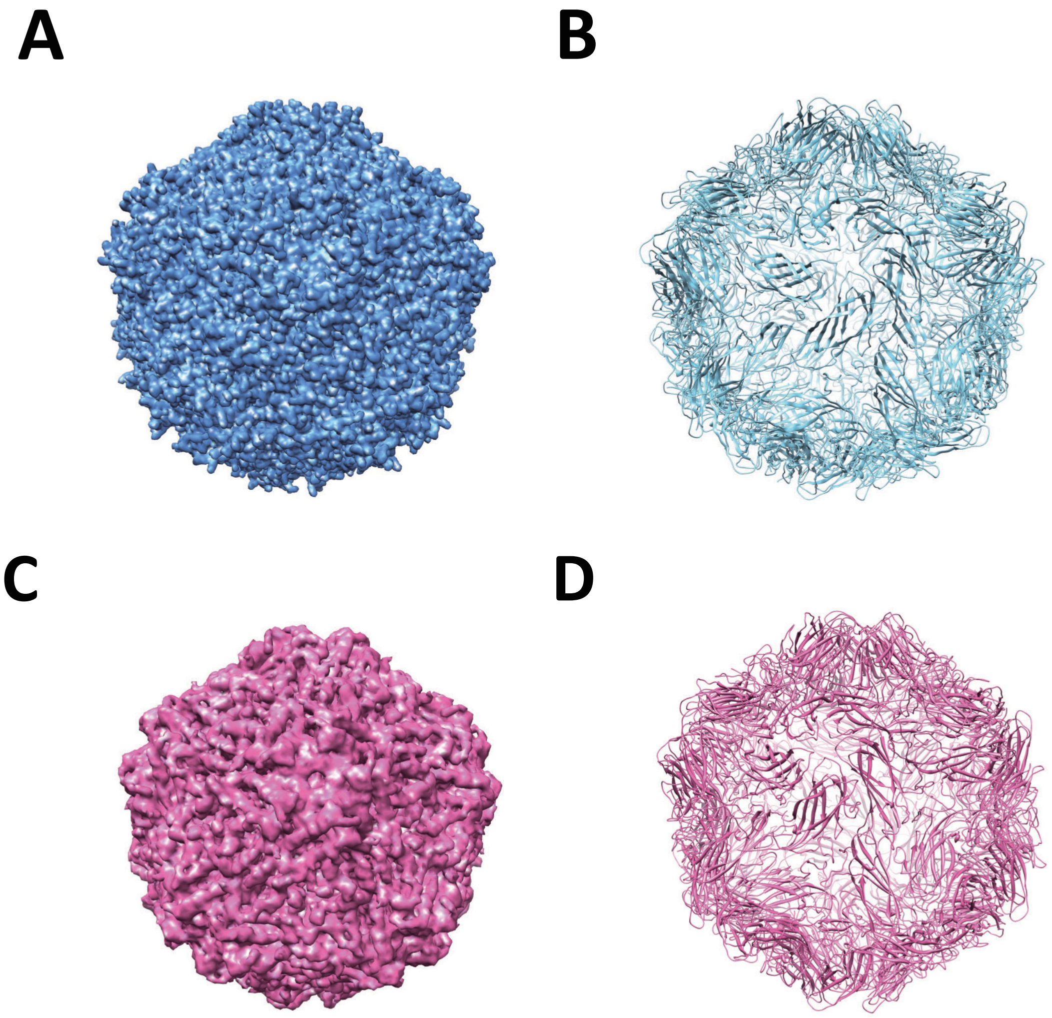

Structural comparison of PCV2 VLPs determined by either cryo-EM or X-ray crystallography approaches
