# Supplementary material for: Structural roles of PCV2 capsid protein N-terminus in PCV2 particle assembly and identification of PCV2 type-specific neutralizing epitope
Source: PLoS Pathog. 2019 Mar 1;15(3):e1007562. doi: 10.1371/journal.ppat.1007562 (PMC6415871; doi:10.1371/journal.ppat.1007562)
Supplement: S3 Fig — (A) PCV2 VLP slices through input volume. (B) PCV2 VLP slices through ResMap results. (C) Histogram of PCV2 VLP ResMap results. (D) Refined cryo-EM structural model of full-length PCV2 VLP reconstructed at 4.12Å. The structural details of PCV2 at the surface loop regions are enlarged and shown. (PDF) [file ppat.1007562.s004.pdf]

S3 Fig. Mo et al.

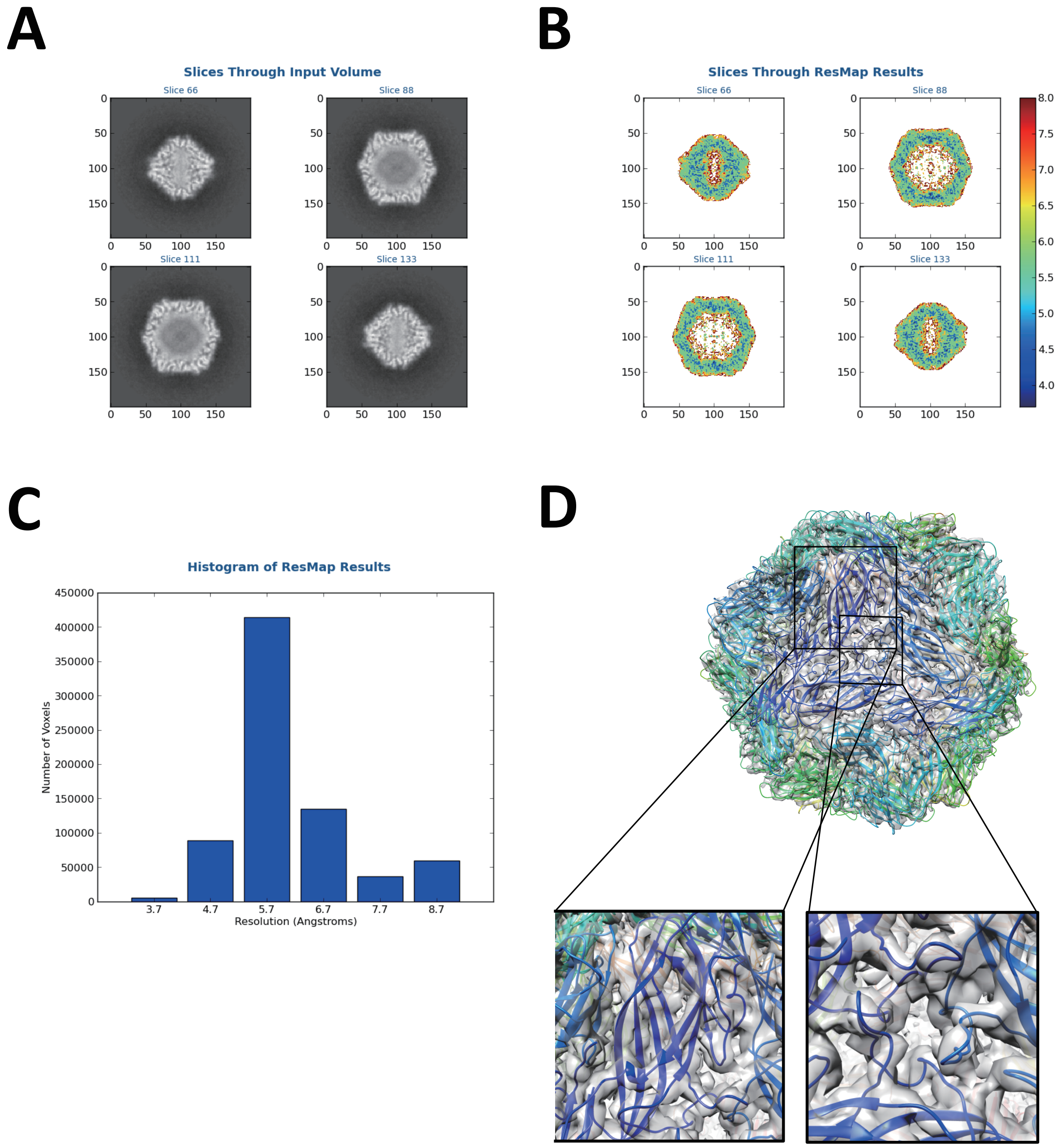

Local resolution assessment of reconstructed 3D cryo-EM structure of full-length PCV2 VLPs
