# Supplementary material for: Structural roles of PCV2 capsid protein N-terminus in PCV2 particle assembly and identification of PCV2 type-specific neutralizing epitope
Source: PLoS Pathog. 2019 Mar 1;15(3):e1007562. doi: 10.1371/journal.ppat.1007562 (PMC6415871; doi:10.1371/journal.ppat.1007562)
Supplement: S5 Fig — (PDF) [file ppat.1007562.s006.pdf]

S5 Fig. Mo et al.

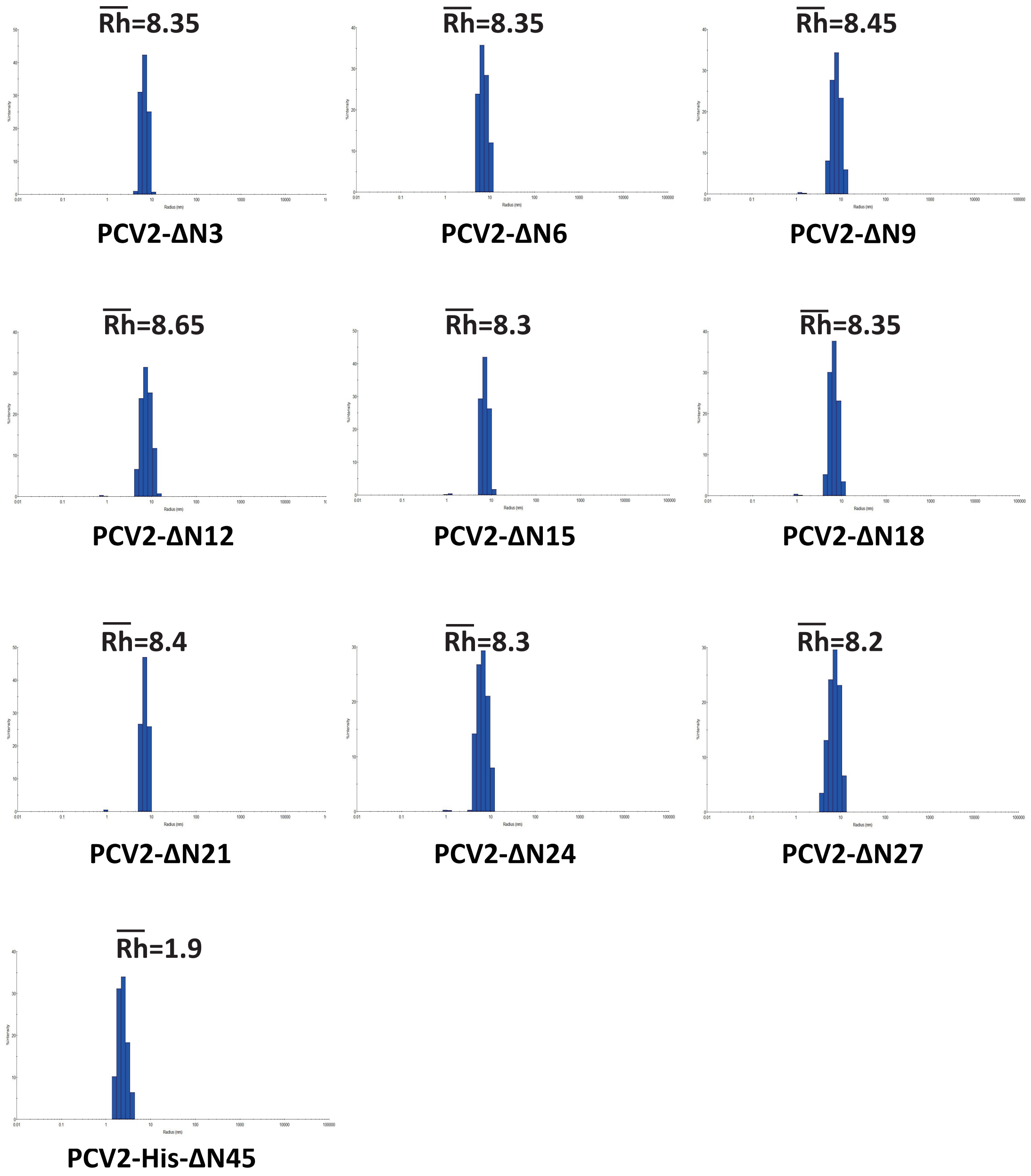

Dynamic light scattering measurement of truncated PCV2 capsid proteins in VLP assembly
