# Supplementary material for: Structural roles of PCV2 capsid protein N-terminus in PCV2 particle assembly and identification of PCV2 type-specific neutralizing epitope
Source: PLoS Pathog. 2019 Mar 1;15(3):e1007562. doi: 10.1371/journal.ppat.1007562 (PMC6415871; doi:10.1371/journal.ppat.1007562)
Supplement: S8 Fig — (PDF) [file ppat.1007562.s009.pdf]

S8 Fig. Mo et al.

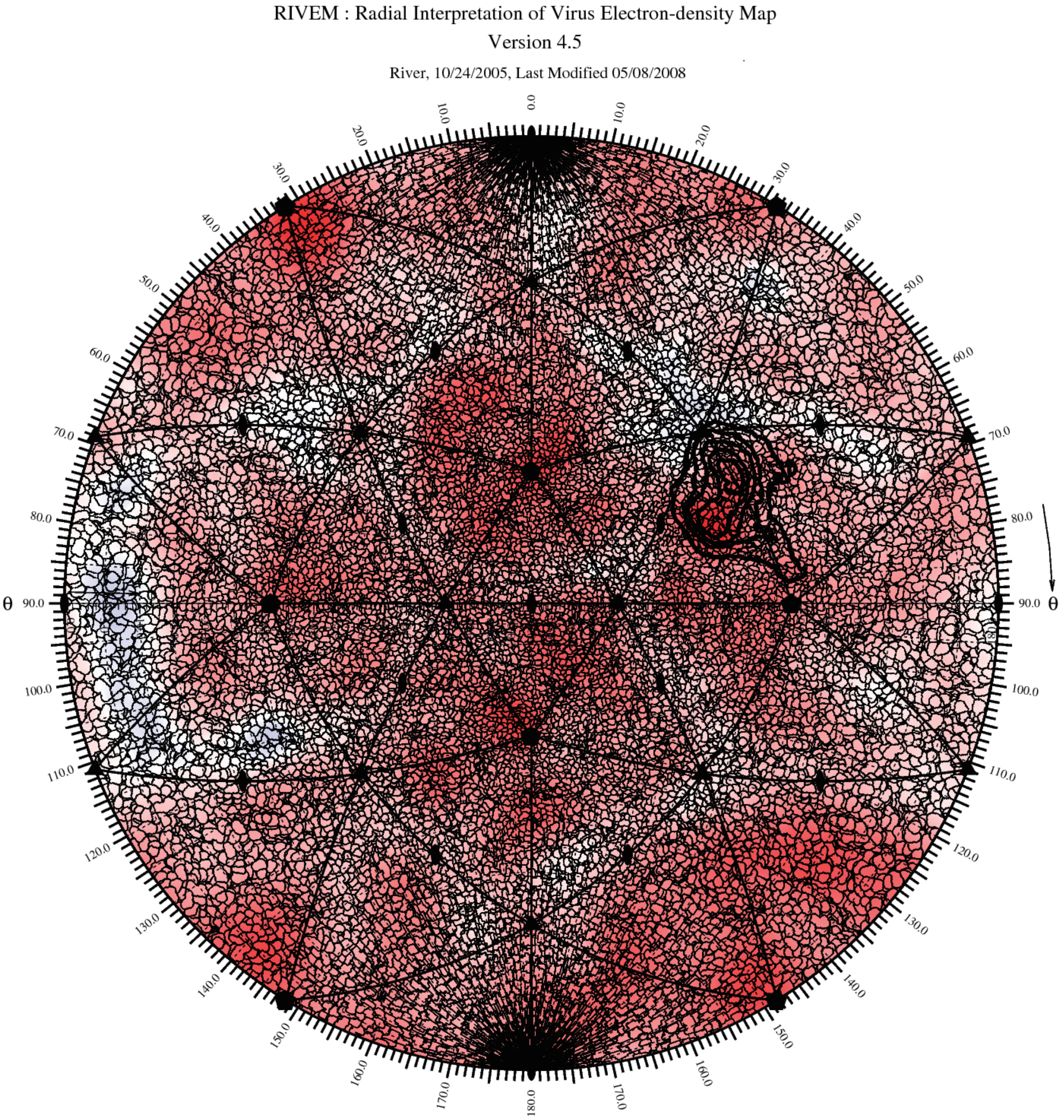

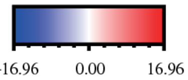


```
Input information:

Inputted Map:
 Dimensions :    Column   Row   Section
      Whole :     160     160     160
     Actual :     160     160     160
      Start :     -80     -80     -80
      End   :     160     -80      79
  Cell      : 435.680 435.680 435.680
               90.000  90.000  90.000
 Voxel Size :     2.723
 Map Min Max:   -60.169     64.013
 Map Avg Std:    10.139      8.236
Inputted PDB:
 Input PDB file: PCV2_3H11.pdb
 Number of Atoms: 238055
 X range: -224.157 -  224.157
 Y range: -224.157 -  224.157
 Z range:   -0.190 -  224.157
 Atom Radius Range:     1.700 -     3.264
 R range:    2.286 -  238.588
 Theta range:   0.04 - 179.96
 Phi   range:   0.00 - 180.05

Inputted Matrices
```

```
Plot Ranges:

 The R max limit is      : 217.840004
 The R step size is      : 2.723000
 The Phi min limit is    : 0.000000
 The Phi max limit is    : 180.000000
 The Theta min limit is  : 0.000000
 The Theta max limit is  : 180.000000
 The angle step size is  : 0.100000

Output information:

Output Filename: _.ps
Output Map:
 Output electron density map in the limit range:
 Density Range:   -2.841 -    16.961
 Density Averge:     5.703
 Density R.M.S.D.:     2.949

Spherical Roadmap:
 Number of Atoms: 7143823
 X range: -224.157 -  224.157
 Y range: -224.157 -  224.157
 Z range:   -0.190 -  224.157
 Atom Radius Range:    1.700 -     3.264
```

Plot Range of Modeling coordinates of cryo-EM structure of PCV2 VLP+ 3H11 Fab complex by RIVEM
