# Supplementary material for: Structural roles of PCV2 capsid protein N-terminus in PCV2 particle assembly and identification of PCV2 type-specific neutralizing epitope
Source: PLoS Pathog. 2019 Mar 1;15(3):e1007562. doi: 10.1371/journal.ppat.1007562 (PMC6415871; doi:10.1371/journal.ppat.1007562)
Supplement: S11 Fig — (PDF) [file ppat.1007562.s012.pdf]

S11 Fig. Mo et al.

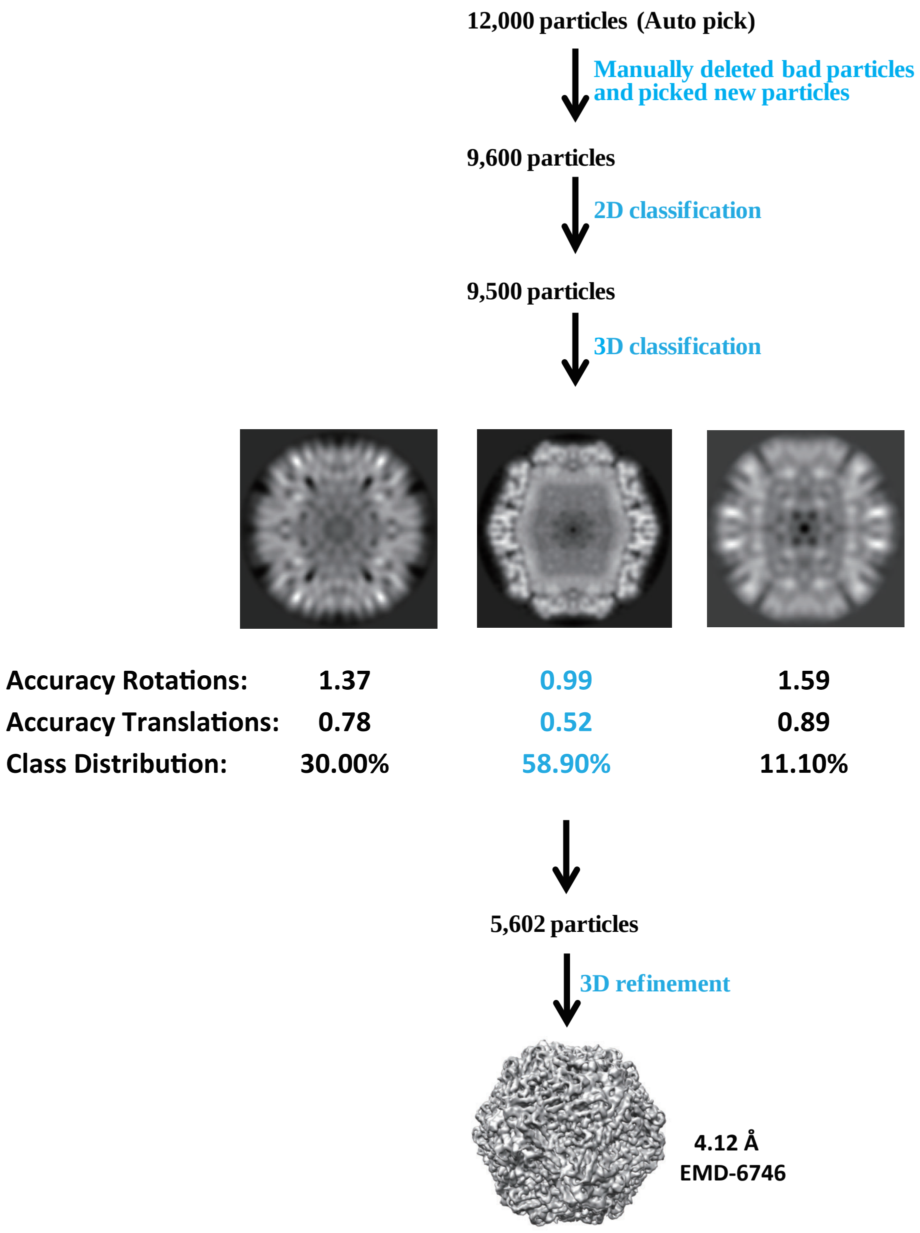

A flow-chart for the cryo-EM data processing of the full length PCV2 VLP. Please refer to the Method for details.
